# Sea Cucumber Derived Type I Collagen: A Comprehensive Review

**DOI:** 10.3390/md18090471

**Published:** 2020-09-18

**Authors:** Tharindu R.L. Senadheera, Deepika Dave, Fereidoon Shahidi

**Affiliations:** 1 Department of Biochemistry, Memorial University of Newfoundland, St. John’s, NL A1B 3X9, Canada; trlsenadheer@mun.ca; 2 Marine Bioprocessing Facility, Centre of Aquaculture and Seafood Development, Fisheries and Marine Institute, Memorial University of Newfoundland, St. John’s, NL A1C 5R3, Canada

**Keywords:** sea cucumber, collagen, characterization, physicochemical properties, applications

## Abstract

Collagen is the major fibrillar protein in most living organisms. Among the different types of collagen, type I collagen is the most abundant one in tissues of marine invertebrates. Due to the health-related risk factors and religious constraints, use of mammalian derived collagen has been limited. This triggers the search for alternative sources of collagen for both food and non-food applications. In this regard, numerous studies have been conducted on maximizing the utilization of seafood processing by-products and address the need for collagen. However, less attention has been given to marine invertebrates and their by-products. The present review has focused on identifying sea cucumber as a potential source of collagen and discusses the general scope of collagen extraction, isolation, characterization, and physicochemical properties along with opportunities and challenges for utilizing marine-derived collagen.

## 1. Introduction

During the past few decades, sea cucumbers and other marine invertebrates have been exploited for commercial use for food and health purposes. Recently, seafood and its derivatives have become one of the most traded food commodities around the globe for disease risk reduction and health promotion [1,2,3]. The trend further enhanced the popularity of this marine species among the scientific community as well as consumers [4,5]. Apart from the high nutritional profile of sea cucumber, the availability of unique bioactive compounds coupled with the therapeutic properties has upgraded their position as a functional food ingredient. Bioactive compounds including antioxidant, antihypertensive, anti-inflammatory, anticancer, antimicrobial, and anticoagulant/antithrombotic compounds have been identified in different sea cucumber species available around the world [2,6]. Moreover, sea cucumbers play a vital role as echinoderms in the marine ecosystems and are primary organisms comprised of grazing, predation and bioturbation in benthic areas and deep oceans across the world [7]. Hence, it becomes essential to utilize these marine resources sustainably with consideration of environmental and commercial perspectives.

The versatile nature of the unique functional and technological properties of sea cucumber has extensively been studied and has shown great potential for developing novel foods as well as bio-medicinal applications [8,9,10]. The groups of bioactive compounds identified with elucidated structures are collagen, gelatin, saponins, chondroitin sulphates, glycolipids, triterpene glycosides, mucopolysaccharides, bioactive peptides, vitamins, minerals, carotenoids, and amino acids, among others [5,11,12,13]. Within these numerous bioactive compounds, sea cucumber has been considered as a rich source of collagen [14]. As soft-bodied marine invertebrates, sea cucumbers possess leathery skin and an elongated body with the body wall attaining the highest market demand [15]. Recently, sea cucumbers have gained increased attention as one of the primary sources for high-quality marine collagen as alternatives to mammalian collagen due to the recent occurrences of pathogenic diseases and religious sentiments [8,16,17]. However, the amount and the nature of collagen available in sea cucumbers depend on the species, biological environment, and diet. The most recent study on *Cucumaria frondosa* demonstrated that extractable collagen from its body wall is less than a fraction of one percent [18]. This could be related to the feeding habit of *Cucumaria frondosa* that is associated with the phytoplankton, zooplankton, and other organic matter whereas other species feed on mud and dead particles on the sea floor.

Considering the collagen as a biomaterial, it has many usages in several fields [19]. Application of collagen is diversified mainly due to its unique properties such as biocompatibility, low antigenicity, high biodegradability, and cell growth potential [17]. Apart from the food industry, collagens have been widely used in tissue engineering, pharmaceutical, and biomedical industries as well as various other fields, including cosmetics [20]. For these applications, the quality and purity of collagen play a significant role. Besides the origin of raw material, extraction conditions might have a direct influence on the yield and properties of the resultant collagen. The extracted crude collagen requires downstream processing for purification based on the quality of crude collagen and is subsequently available for a wide range of applications covering both medical and industrial domains.

Furthermore, to obtain high-quality collagen, it is crucial to consider pre-treatment steps prior to extraction procedures. In addition, paying attention to the recovery of collagen is beneficial, both from the isolation and purification methodology standpoints [10]. In addition, characterization of the extracted collagen is another key factor that helps identifying its potential as a biomaterial in diversified applications. Therefore, exploring novel approaches to extract the collagen from sea cucumber with less environmental impact and studying their application areas have attracted the interest of researchers from both scientific and industrial communities. The present review provides some background information about collagen and explains its different sources including that from sea cucumber with emphasis on the extraction, isolation, purification, and characterization techniques mainly related to type I collagen. In addition, use of collagen and its functional properties as well as challenges and future perspectives of utilizing collagen from sea cucumbers are discussed.

## 2. Definition and History of Collagen

As one of the most abundant fibrous proteins, collagen plays a vital role in connective tissues, thus animal skin and bone provide an extracellular framework for strength and flexibility [9]. Collagen is one of the major structural proteins in the extracellular matrix and the name is derived from the Greek word “kola,” which means “glue producing”. Moreover, “collagen” is considered a generic term, and no well-defined criteria exist to name this structural biopolymer [21,22]. Findings of Schweitzer et al. [23] revealed the presence of intact collagen in the soft tissue of the fossilized bones of 68 million-year-old *Tyrannoaurus* rex, a genus of *coelurosaurian* theropod dinosaur. Sequences of studies have been conducted for decades to propose a structure for the collagen molecule. Among those studies, triple-helical “Madras Model” by Ramachandran and Kartha [24] contributed much to the currently accepted structure of collagen which was discovered by Cowan, North and Randall [25]. Further findings of Rich and Crick [26] also improved the identified structure of collagen [27]. Currently, more than 29 distinct types of collagen have been identified [28,29].

The molecular structure of collagen consists of three polypeptide α chains intertwined with each other to form a triple helix, approximately 300 nm in length with a molecular weight of 105 kDa [8]. These molecules can either be homomeric (contain identical α chains) or heteromeric (genetically distinct α chains) [30]. Each strand is initially shaped into a left-handed symmetry prior to their conformation as a right-handed triple helix.

**Figure 1:**
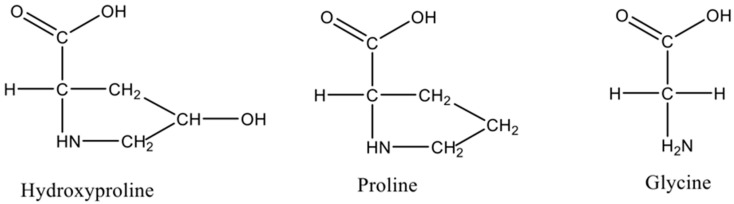
Amino acid residues present in triple helix.

**Figure 2:**
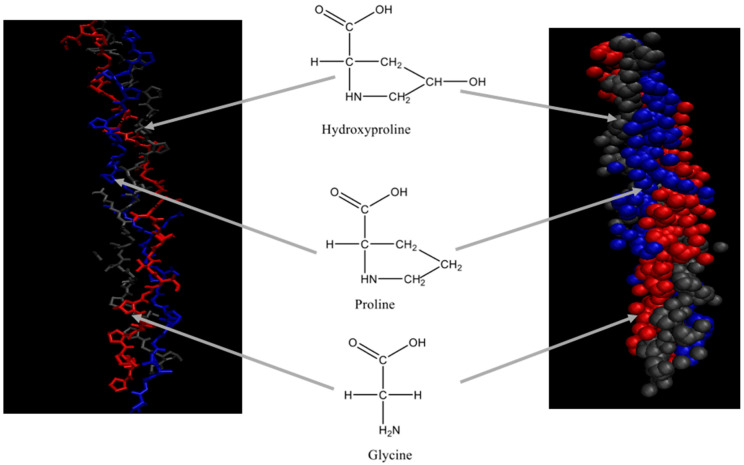
Triple helix structure of collagen.

Each chain of the right-handed helical structure consists of a repeating sequence of glycine-X-Y, where often, X and Y are referred to proline or hydroxyproline, respectively (Figure 1) [29]. In this motif, all glycine residues are located inside the core, while other amino acids (X and Y) are located on the surface [30]. This rigid rod-like structure is further strengthened by interchain N-H (Gly) O=C(x) hydrogen bonds and electrostatic interactions [31]. The presence of triple helix (Figure 2) is the main feature in the collagen structure. However, triple helix can be varied according to the type of collagen present in the structure [32]. Moreover, this sequential uniformity can rarely be found in other proteins. Due to the uniformity of collagen, numerous studies have been conducted to determine their potential as a prospective biomaterial for a wide range of applications.

### 2.1. Basic Structure and Synthesis

The fundamental subunit of collagen is tropocollagen which is a three-stranded polypeptide unit. Collagen family is classified into various groups due to their complex structural diversity [21]. Different lengths of the helix, presence of non-helical components, interruptions in the helix, variations in the assembly of the basic polypeptide chains, and differences in the terminations of the helical domain directly lead to distinct types of collagens. Its general groups include fibrillar collagens, FACIT (Fibril Associated Collagens with Interrupted Triple Helices), FACIT-like collagen, beaded filament collagen, basement membrane collagen, short-chain collagen, transmembrane collagen, and unclassified collagen [29,33]. The length of the helix and portions of non-helical components are different depending on the type of collagen.

Numerous studies on collagen have revealed that collagen type I is the most ubiquitous form of collagen which belongs to the fibrillar group [29,30]. Fibril forming collagen or fibrillar collagen is synthesized in the form of soluble precursor molecules (procollagen) by the process of fibrillogenesis. Each polypeptide chain is involved in the synthesis process and consists of N- and C- propeptides at each terminal position of the triple helix [22]. The fibrils produced have a visible banding, a direct result of the aggregating pattern of collagen. The stability of the fibrillar collagen depends on non-reducible covalent cross-links in the triple helix [34].

The name FACIT collagen implies that the association of fibrils is interrupted by non-helical domains. They are linked with the surface of collagen fibrils and the collagenous structure is disturbed by non-helical domains. Wang et al. [22] further described that the C-NC domain in FACITs is short compared to fibril forming collagens. Collagen types IX, XII, XIV, XVI, XIX, XX, XXI, and XXII belong to the FACIT group [32]. Wu, Woods, and Eyre [35] explained this scenario by depicting the structure of type IX collagen that lies anti-parallel to type II fibrils. Moreover, primary sequences of some FACIT collagens share similarities with fibrillar collagens [29].

The beaded filament collagen molecules assembled without undergoing the cleaving of terminal regions and the formation of the bead region in collagen filaments are facilitated by these uncleaved regions [29,32,35]. The most characteristic feature of this subgroup is having large N- and C-terminals. For example, type VI is having large N- and C-terminals even in their short triple- helical domains [29,30,36]. Furthermore, only type VI collagen belongs to the subgroup of beaded filament collagen [29].

The basement membrane and associated collagen are categorized under non-fibrillar collagen. They can be found mostly in tissue boundaries, which facilitate molecular filtration by forming a connected network, especially in basement membranes [21,22]. Apart from tissue boundaries, non-fibrillar collagen can be found in cavities of the epithelial lining, endothelium in the interior blood vessels, fat, muscle, and nerve cells. Based on the electron microscopic images, collagen IV belongs to the non-fibrillar collagen subgroup that appear as thin sheets and its molecules are relatively long compared to the fibrillar collagen [30]. Anchoring fibrils collagen VII are considered as essential for functional integrity [22]. Short-chain collagens are described as mesh forming collagen and are located in underlying endothelial cells. Some of the short-chain collagens are also present in mineralizing cartilage [28]. The short-chain collagen possesses a shorter triple-helical region (half of the length of fibrillar collagen). Type VIII and X are categorized under the subgroup of short-chain collagen. Among them, type VIII collagen involves the proliferation of cells as a growth enhancer [29].

The transmembrane collagens function as cell surface receptors as well as matrix components involved in adhesion [29,30,37]. Moreover, they possess a relatively long but interrupted triple-helical domain with a short N terminal domain [38]. Type XIII, XVII, XXIII, XXV, and other collagen-like proteins are categorized under transmembrane collagens [37,38].

### 2.2. Nomenclature, Types, and Classifications

After discovering type I, II, and III collagen, further research studies were evoked on the identification of possible molecular types of collagen. However, expanded studies indicated that type III collagen molecules also contained type I collagen and both types together could form mixed fibrils. This observation affected the terminology that existed then and became more complicated after the identification of type IV collagen [39]. Due to the variations of their histology, it was agreed to give a type number so, they are numbered with Roman numerals (I-XXIX) while polypeptide chains are named using α chains with Arabic numerals (α1, α2, α3, etc.). For instance, type I collagen with identical α1(I) chains and one chain α2(I) and the nomenclature for type I collagen is [α1(I)]_2_ α2(I) [38,39]. Table 1 represents the some of the common types of collagen with their nomenclature and distribution.

## 3. Sources of Collagen

**Figure 3:**
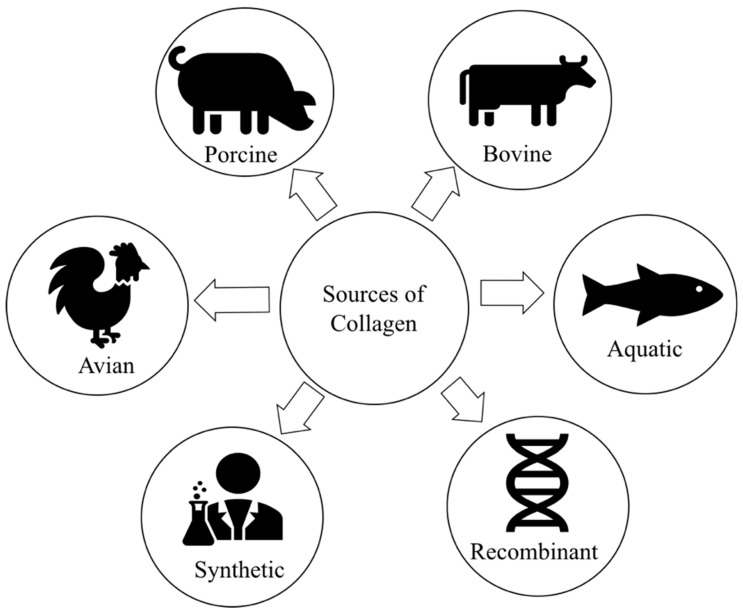
Popular sources of collagen.

As the major structural proteins are in the skin and bones of most animals, collagen accounts for 30% of the total body protein [10]. The most common raw materials for collagen production are obtained from the slaughterhouse by-products, including hides, bones, tendons, and cartilages, or recombinant collagen. At the industrial-scale production, animals such as bovine and pigs are used as primary sources of collagen [9]. Figure 3 represents the most common sources of collagen. However, the outbreak of prion diseases such as bovine spongiform encephalopathy (BSE) resulted in some barriers for using bovine collagen whereas swine flu has limited the use of porcine collagen [42].

In addition, due to various religious constraints, porcine or mammalian collagen for the development of kosher and halal products is limited [10,21,22]. Apart from the widely used species, several studies (Table 2) have extracted collagen from chicken [43], kangaroo tail [44], rat tail tendon [45], duck feet [46], equine tendon [33], alligators bone [47], birds’ feet [48,49,50], sheep tendon [51,52,53,54,55], and frog skin [56], while some studies have focused on using recombinant human collagen [20]. The high pathological risk for transmitted diseases and complicated extraction process have limited the applicability of using land animal collagen and created a growing concern towards finding alternative sources for collagen. The two primary sources of industrial collagen, including land animal by-products and marine organisms, are described in the following subsections.

### 3.1. Land Animal By-Products

In recent decades, inedible animal by-products are utilized to produce fertilizers, minerals, fatty acids, vitamins, protein hydrolysates, and collagen [62]. Bovine collagen is the primary source for the industrial collagen used in medicine, cosmetics, and other non-biomedical applications [63]. Sterilized purified collagen from cow skin is used as injectable bovine collagen [64]. Apart from BSE risk, around 3% of the population is allergic to bovine collagen, which hinders its usage [20].

Skins and bones of pigs are used to extract porcine collagen [20]. Pig rind is famous for processing food products such as sausage casings and edible films. Moreover, porcine collagen is used as a dermal substitute in the medical field as they are used widely as implants for reconstructive surgery [65]. Pig hides are used to extract porcine type I collagen and share similar properties to human collagen, hence it has a wide range of application in both medical and food industries [65,66,67].

Collagen extraction from poultry by-products such as skin, bones, and cartilage from chicken has also been reported. However, the usage was limited due to the occurrence of avian influenza [68]. The mammalian collagen is preferred in the industrial level applications over avian collagen. The limited applications of avian collagen correlate with the expensive and complicated extraction process.

### 3.2. Marine Organisms

**Figure 4:**
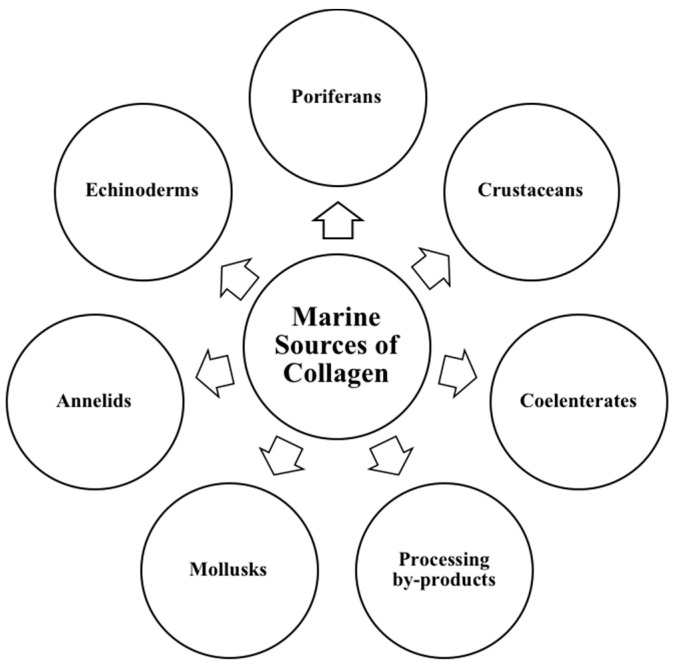
Marine sources of collagen.

The marine-derived collagen is a promising alternative due to the occurrence of foot-and-mouth disease (FMD), BSE, and avian influenza like diseases, as well as religious and social constraints [69,70]. Several comprehensive reviews on marine-derived collagen and their application in various fields have appeared [17,20,71]. Recently, collagen from various marine organisms such as poriferans, coelenterates, annelids, mollusks, echinoderms, and crustaceans has been extensively investigated (Figure 4).

The unique characteristics of marine collagen as a biomaterial with significant biocompatibility and biodegradability has been favored in many industrial applications over other alternate sources [69,72]. Mainly, marine by-products have been exploited to recover collagen and other collagen-derived biomaterials through a combination of different bioprocessing methods [73]. These include Japanese sea bass skin [74], clown feather back skin [75], bladder of yellow fin tuna [76], fin, scales, skins, bones, and swim bladders of big head carp [77], skin and bone from Japanese seerfish, cartilage from sturgeon and sponges, sea urchin [78], octopus [79] squid [80], cuttlefish [81], sea anemone [82], and sea cucumbers for extraction of marine collagen [83]. Particularly, collagen type I was extracted from the skin of silver carp [84], Japanese sea-bass [74], mackerel [85], bullhead shark [86], and sole fish [87] as well as from the bones of skipjack tuna [88], and scales of Nile tilapia [89].

Significant differences in the amino acid composition of collagen from various fish species are responsible for their unique characteristics [69]. Most of the fish collagen contains a lower proportion of hydroxyproline compared to mammalian and avian collagen. Consequently, their lower compatibility to crosslinking and stability compared to other types of collagen has been reported [14,69,90]. However, the content of hydroxyproline also depends on the habitat of fish species [91]. Moreover, the thermal stability of the collagen extracted from warm water species is found to be higher than cold water species [91].

The marine sources of collagen have received increasing attention due to their availability, easy processing techniques, safety (free of zoonosis), environmentally friendly extraction procedures, low molecular weight, less religious and ethical barriers, minor regulatory and quality control problems, a negligible amount of biological contaminants and toxins, low inflammatory response and excellent metabolic compatibility [20]. However, most studies have been conducted to identify the potential uses of collagen derived from marine vertebrates but reports on marine invertebrates are scarce [4,17]. Thus, current research interest is directed towards the use of marine invertebrates as potential sources of collagen, particularly for biomedical applications. Recent investigations have been concentrated on jellyfish [92] sponges [93], mussels [4,94], and sea cucumber [14,16,83,95,96,97,98,99,100,101,102,103,104,105] as potential candidates for producing marine-derived collagen.

### 3.3. Sea Cucumber as A Source of Collagen

Among the various bioactive compounds derived from sea cucumber, collagen plays a vital role. Primary intensive research on sea cucumber collagen has been initiated in the early 1970s by Eyre and Glimcher [106] and Matsumura, Shinmei, and Nagai [107]. Eyre et al. [108] studied the comparative biochemistry of the collagen crosslinks using sea cucumber *Thyone briarius*, a sponge *Haliclona oculata*, and sea urchin *Strongylocentrotus droebachensis* and reported the evidence for glycosylated crosslinks in collagen derived from the body wall of sea cucumber Thyone *briarius*. Matsumura et al. [107] then focused on the purification of collagen from sea cucumber *Stichopus japonicus* by disaggregating the connective tissue of body wall followed by the morphological study of the isolated collagen fibrils. Furthermore, the most extensive research studies were focused on the molecular structure and functional morphology of *Cucumaria frondosa* which led to a series of discoveries on the covalent composition and growth of collagen fibrils in the same species [109,110,111]. The dermal glycoprotein stiparin was identified as the main factor responsible for the aggregation of collagen fibrils from the dermis of sea cucumber *Cucumaria frondosa* [112] and Trotter et al. [113] characterized a sulphated glycoprotein, which inhibited fibril-aggregating activity.

Thurmond and Trotter [114] further investigated the morphology and biomechanics of the microfibrillar network of collagen derived from sea cucumber *Cucumaria frondosa* dermis and reported similar morphological characteristics with fibrillin microfibrils of vertebrates. Most of the early investigations of the sea cucumber collagen fibrils contributed to recent developments of the research related to collagen and other bioactivities from sea cucumber. Table 3 provides a cursory account of recent studies related to sea cucumber collagen.

Sea cucumber research interests have been mainly focused on cultivation and bioactive molecules. Most of the research conducted on bioactive ingredients from sea cucumber has centered around proteoglycan and collagen [83]. The main edible portion of sea cucumber is the body wall composed of mutable connective tissue (MCT) with scattered cells [114]. The structural components of MCT consist of collagen, proteoglycan, and glycoprotein [118]. These assembled components form collagen fibrils, collagen fibers, and microfibrils. Among them, the majority of total body wall protein are comprised of insoluble collagen fibrils. Collagen fibers are surrounded and separated from the microfibrillar network in MCT and this network maintains the organization while providing a long-range restoring force [120].

The most abundant type of collagen found in sea cucumber is type I collagen and collagen fibrils of echinoderms are symmetrically spindle-shaped and short in length [113,120]. Moreover, at the molecular level, they are considered as bipolar collagen fibrils with surface associated proteoglycans [113]. Covalent crosslinks providing stabilization to collagen are internally present and similar to the mammalian collagen. Besides, absence of permanent crosslinks in the structure improves the isolation of collagen fibrils in their intact form [98,113]. It also helps to slide pass one another during lengthening and shortening of the tissue [114]. The solubilized collagen from the body wall of sea cucumber (*Stichopus japonicus*) has distinct subunit structure of (α1)_2_ α2 and are rich in glutamic acid. Thermal denaturation of this type of collagen may impart unique textural properties [14]. A recent study on the molecular composition of collagen fibrils isolated from sea cucumber *Aposticopus japonicus* revealed that collagen fibrils are heterotypic containing two clade A, one clade B fibrillar collagens, and two FACIT collagens [104]. Fibrillar collagen α chains may be classified in to three clades according to their evolutionary steps. Clade A consists of α1(I), α2(I), α1(II), α1(III), and α2(V) chains; clade B contains α1(V), α3(V), α1(XI), and α2(XI) chains while clade C includes α1(XXIV) and α1(XXVII) chains [129].

Tian et al. [104] also reported the heterogenicity exhibited in the pepsin-solubilized collagen isolated from *Aposticopus japonicus* for the first time. Their novel findings on subunit compositions and constituents of sea cucumber collagen were, however, contradictory to the previous studies [14,16,98,99,101,102,109,115,120]. Most of the previous studies focused on pepsin-solubilized collagen (PSC), and structure analysis was conducted using sodium dodecyl sulphate polyacrylamide gel electrophoresis (SDS-PAGE). However, Tian et al. [104] used proteomic techniques and bioinformatic methods to analyze the constituents present in sea cucumber collagen. According to the phylogenetic analysis of identified collagen sequences revealed that reported sea cucumber collagen sequences did not belong to the branches of typical collagens. The authors concluded that the heterogenic and complex nature of the sea cucumber collagen is complicated and needs extensive investigations. Thus, previously reported studies on SDS-PAGE analysis are not considered adequate to conclude the fundamental molecular structure of collagen [104].

## 4. Characteristics and Properties of Collagen Type I

**Figure 5:**
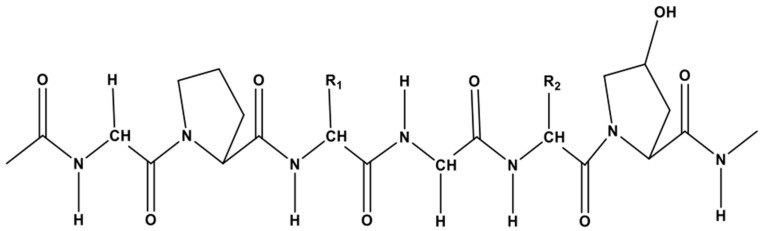
Chemical structure of collagen type I-Primary amino acid sequence.

Hierarchical structures configured from the fibrillar collagen include collagen α chains, tropocollagen, collagen fibril and collagen fibers [104,130]. The most abundant structural collagen in most tissues is the fibrillar type I collagen [131]. Primarily, type I collagen (Figure 5) is present in fibril surface as well as notably in connective tissues of the skin and bone and has distinct structural features including wide distribution of fibril diameters and high internal crystallinity [64]. Type I collagen fibril is formed by two equivalent α1 and one α2 polypeptide chains and composed of 1.1 × 300 nm size collagen molecules [130,132]. The two α chains form peptide chain dimer referred to as β-peptide chain while three α chains form γ-peptide chain. Each polypeptide chain weighs around 100 kDa and is comprised of 1052 amino acid residues.

The γ-peptide chain is referred to as the tropocollagen molecule. Terminal extensions of type I tropocollagen included 139 amino acids and COOH which weigh 20,000 and 35,000 Da, respectively [41,64,132]. Type I collagen contains high amounts of proline and hydroxyproline compared to other types of collagen [41,132]. Moreover, collagen type I is considered as a glycoprotein and composed of less than 1% carbohydrate, including either single galactose unit or disaccharide of galactose and O-glycosidically attached via hydroxylysine residues as sugar components [41]. Table 4 summarizes some of the distinct characteristics of sea cucumber type I collagen reported in literature compared to the mammalian collagen.

### 4.1. Thermal Stability

The term ‘stability’ in protein ultimately refers to sustaining the significant structure of protein under extreme conditions [133]. The type I collagen is thermodynamically stable compared to kinetic stability [134]. However, the triple helical structure is susceptible to heat and particularly type I collagen has low thermal stability [135]. Moreover, thermal stability is considered as one of the significant factors for determining the potential application of collagen [136]. The amino acid composition directly influences the physical and chemical properties of collagen, including thermal stability. The stability of the triple helix structure is improved by the hydrogen bonds formed by the hydroxyl groups of hydroxyproline [101]. The amino acid content influences the thermal stability of type I collagen. The molecular structure of collagen mainly depends on the secondary structure of the polypeptide chain [137]. Pyrrolidine rings of proline and hydroxyproline are mainly responsible for the unique structure of collagen and the helical stability is directly proportional to the amino acid content [101].

The thermal stability of collagen, as any other protein, is often described in accordance with its denaturation temperature (Td) and the maximum transition temperature (Tm) [101,133]. Td denotes the temperature at which the triple-helix structure of collagen disintegrates into random coils, and when it reaches Tm, half of its triple helix is degraded to obtain the maximum transition temperature of the collagen [101,138]. The transition temperature correlates with collagen stability and durability of collagen-based biomaterials [48]. Determination of the thermal stability using differential scanning calorimetry (DSC) thermogram will be discussed in later sections of this review.

Numerous studies have been conducted to elucidate the thermal stability of isolated collagen from sea cucumber. Thermal behavior of the sea cucumber derived collagen was mostly comparable to type I bovine collagen. Isolated collagen from sea cucumber (*Stichopus monotuberculatus*) exhibited lower (30.2 °C) Tm than calfskin collagen (35 °C) and positive relationship existed among Tm value and imino acid content [101]. Adibzadeh et al. [98] reported a similar trend for the pepsin solubilized collagen from sea cucumber *Holothuria parv* and showed lower thermal stability compared to type I bovine collagen and porcine skin collagen. Thermal behavior of the collagen isolated from *Stichopus japonicus* [16], *Stichopus vastus* [122], and *Parastichopus californicus* [120] further explains the lower transition temperatures irrespective of the species, and shows that sea cucumber derived collagen possesses weak thermal stability compared to mammalian collagen. This may be due to the factors influencing the thermal stability of collagen originating from vertebrates and invertebrates [48]. Besides the amino acid composition (especially amount of amino acid residues), the environment and body temperature of the animal is a determinant factor for thermal sensitivity of collagen fibrils [101]. Lin and Liu [48] revealed that marine collagen has a lower denaturation temperature in contrast with collagen derived from land animals.

It is noteworthy that thermal stability has a direct relation with the amino acid composition as most of the isolated sea cucumber collagen is rich in hydroxyproline and proline [16,101,120]. However, most studies on sea cucumber collagen have been focused on pepsin solubilized collagen (PSC), and these PSCs do not represent the native structure of the dermic collagen [138]. Qin et al. [138] studied the thermal behavior of insoluble collagen fibrils and PSC from sea cucumber (*Stichopus japonicus*). According to their findings, helical structures of insoluble collagen fibrils are more stable than those of pepsin soluble collagen. The difference is mainly due to the removal of cross-linkages in the telopeptide region of native collagen fibrils. Therefore, insoluble collagen fibrils show higher thermostability compared to PSC [138].

Furthermore, thermal stability plays a significant role in the sea cucumber processing industry [117]. Few studies have examined the thermal behavior of collagen during processing. One study investigated the thermal denaturation of crude collagen fibers (CCF) and PSC during cooking [115]. CCF was more thermostable than the PSC at different tested cooking temperatures of 40–100 °C. Besides the DSC method, a Fourier transform infrared (FTIR) method was also employed to analyze the thermal properties of collagen. Si et al. [117] combined the FTIR and thermogravimetric analysis (TGA) to determine the thermal degradation mechanism of sea cucumber (*Stichopus japonicus*). The thermal degradation activation energy of sea cucumber collagen revealed that higher treatment temperatures were not applicable for cooking or processing of sea cucumbers. In addition, it may have adverse effects on the nutritional value of sea cucumber due to the denaturation of proteins [117].

Furthermore, previous studies have reported the effect of thermal treatment on collagen fibrils in relation to oxidation [139]. A recent study investigated the molecular structure of collagen isolated from *Apostichopus japonicus* during thermal treatment in the presence of (-)-epigallocatechin gallate (EGCG) [105] and demonstrated that EGCG has the potential to enhance the thermal stability of crude collagen fibrils by neutralizing the effect of heat-induced radicals (hydroxyl radical) and protect the macromolecular structure of crude collagen in a dose-dependent manner [105].

### 4.2. Enzymatic Resistance and Digestion

The biomaterial market prefers a higher enzymatic resistance collagen due to its higher durability [48]. Enzymes that could break the triple helix of collagen are known as collagenolytic enzymes [140]. As collagen plays a role as one of the primary structural body proteins, it has peculiar resistance for neutral proteases [41]. Degradation of collagen molecules starts from the exterior by binding of collagenase to the triple helix near the surface and proceeds with the progression of degradation in the interior of molecules when exposed to the collagenase enzymatic action [41]. Several studies have indicated that collagenase is capable of cleaving all three α- chains of type I collagen at a single site and results in the formation of fragments about three quarters and one-quarter of the original size of the molecules [140].

Liu et al. [119] investigated the role of collagenase type I on the structural features of collagen fibers from sea cucumber (*Stichopus japonicus*). Collagenase partially depolymerized collagen fibers into fibrils of *Stichopus japonicus* by influencing proteoglycan interfibrillar bridges. Furthermore, collagenase has the potential of degrading the monomeric collagen [119]. These findings provide evidence about the role of collagenase in the autolysis of sea cucumber. The autolysis of sea cucumber is due to an activation process of endogenous proteinases such as cysteine proteinase, serine proteinase, and matrix metalloproteinases. Proteases responsible for autolysis are involved in the depolymerization and unfolding of collagen fibrils [141,142]. Collagenase enzyme represents the matrix metalloproteinases group, and collagenase from the dermis of *Stichopus monotuberculatus* was reported to hydrolyze the triple-helix of collagen [142]. These observations are in accordance with the conclusions of Liu et al. [119], implying that endogenous matrix metalloproteases have the ability of digest the macromolecular and monomeric collagens from sea cucumber.

Moreover, serine collagenases are considered as specific collagenase enzymes that can break down the substrate under any conditions. After the initial cleavage of collagen, the polypeptide chains are further degraded by other protease enzymes such as gelatinases and non-specific proteinases [41]. Trypsin belongs to the group of a serine protease and used as a hydrolyzing agent to determine the role of serine proteases in the autolysis process of sea cucumber collagen [118]. The results showed that trypsin has the potential to partially disintegrate the collagen fibrils as well as cleave the interfibrillar proteoglycan bridges with a lower effect on monomeric collagen. Liu et al. [102] studied the effect of endogenous cysteine proteinases on collagen fibers from *Stichopus japonicus* and reported changes in the microstructure of collagen fibrils. Endogenous cysteine proteinases degraded the interfibrillar proteoglycan bridges and increased the structural disorder of fibrillar collagen. Investigations of cysteine proteases, including cathepsins K and L, demonstrated the disintegration of the collagen fibrils caused by the activity of cathepsin L-proteinase on proteoglycan networks [141].

In contrast, several studies have found that some collagens extracted from different species can have higher stability even in the collagenase solution. Lin and Liu [48] reported that the porcine skin type I collagen was more stable compared to the other collagen species, while Angele et al. [143] indicated the higher stability of equine collagen compared to bovine collagen-based matrix. They concluded that the presence of a higher content of glycosaminoglycan was responsible for the stability of collagen as they blocked the cleavage sites of collagenase. Furthermore, Li and Liu [48] stated that marine animal collagens were efficiently degraded by proteolytic enzymes and more sensitive to non-specific enzymatic hydrolysis compared to land animal collagens.

### 4.3. Isoelectric Point of Collagen

Chemical environment is crucial for the formation of collagen type I fibrils and pH plays a vital role when determining the chemical properties. Proteins have zero electrostatic charges at their isoelectric point (pI) which represents the minimum solubility and maximum precipitation. Thus, pI of the collagen is the primary determinant factor for its solubility. Moreover, pI indicates the pH value which has higher hydrophobic-hydrophobic interaction that leads to precipitation and aggregation of the protein molecules [136].

The pI of the collagen derived from sea cucumbers belongs to the acidic region. Zhu et al. [95] observed the isoelectric point of 4.14 for PSC from sea cucumber *Stichopus japonicus*. Similar findings were reported for PSC from different sea cucumber species including *Stichopus vastus*, pI of 4.67 [122] and *Stichopus monotuberculatus* pI of 4.0 [101]. Furthermore, lower pI values correlated with the type of amino acid residues present in the sample [101]. Most of the reported sea cucumber-derived collagen is abundant in glutamic and aspartic acids [14]. Friess [41] reported maximum collagen degradation at pH 4.4. Furthermore, neutral pH is important for collagenases enzyme to react with the triple helix structure, specifically to cleave the band, which is three quarters away from the N- terminus of the native helix. As the most significant parameter of the protein, the pI is related to the proportion of acidic and basic amino acid residues present in protein [144].

### 4.4. Bioactive Properties of Sea Cucumber Collagen

Marine derived collagen is highly regarded as a valuable source with significant bioactive properties [12,15,100,125]. In terms of sea cucumber collagen, several studies have elucidated its antioxidant potential associated with the radical scavenging capacities. Zhu et al. [95] investigated the pepsin soluble collagen from the body wall of sea cucumber *Stichopus japonicus*. It was demonstrated that hydroxyl radical scavenging ability and DPPH (2,2-diphenyl-1,1-picrylhydrazyl) radical scavenging activity were significantly higher than those of vitamins C and E. The authors concluded that antioxidant activities exerted by sea cucumber body wall was mainly attributed to collagens. Similar findings were reported by Abedin et al. [100] on collagen hydrolysates prepared from sea cucumber *Stichopus vastus* using 2,2-azinobis-(3-ethylbenzothiazoline-6-sulfonic acid) (ABTS) activity assay. The authors also observed Angiotensin I converting enzyme (ACE) inhibitory potential of produced collagen hydrolysates [100]. Abdillah et al. [125] investigated the anti-tyrosine and anti-elastase activities of collagen extracted from body wall of sea cucumber *Holothuria lecospilota* and evaluated its pharmaceutical capacities. The study revealed the efficacy of sea cucumber collagen hydrolysates related to their antiwrinkle capabilities. However, most of the pharmaceutical applications of marine derived collagen has been extensively studied for its potential as a biomaterial. Section 5.1 provides a detailed discussion of biomedical applications of sea cucumber collagen.

## 5. Industrial Applications

**Figure 6:**
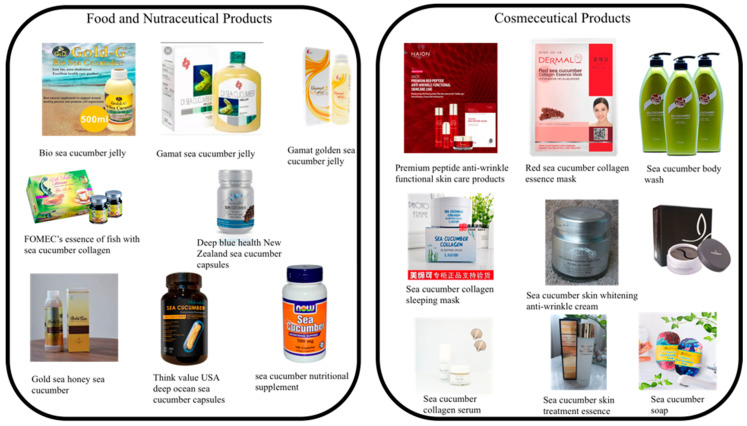
Commercial products developed including sea cucumber collagen as a main ingredient. (Image courtesy: google image; manufactures’ websites).

Collagen may be used in a wide range of applications in various fields due to its diversified nature. The global demand for collagen has increased during the past few decades, with the booming interest for using it as a biomaterial over other natural polymers and their synthetic analogs. Distinct physicochemical properties of collagen expand its application in various fields, including biomedical, pharmaceutical, cosmetic and food industries (Figure 6). Marine collagen, as a promising alternative for commonly used mammalian-derived collagen, has gained growing attention of both scientific and industrial communities. However, reports on the industrial application of sea cucumber collagen are scarce compared to the mammalian collagen. Hence, the following sections include a general overview of potential applications of type I collagen in biomedical and non-biomedical fields.

### 5.1. Biomedical Applications

Collagen is considered as a successful biomaterial in medical applications, mainly due to its characteristics such as biodegradability and weak antigenicity. Its tensile and fibrous structure provides strength and elasticity to the skin in addition to strengthening blood vessels and tissue development [129]. In addition to these properties, the progress of use of collagen as a biomaterial is associated with numerous benefits such as high availability and efficient purification, biocompatibility and bioabsorbability, non-toxicity, synergism with bioactive compounds, compatibility with synthetic polymers, durability and persistence, ability for interaction with cell-matrix and platelets and most importantly fibril reformation [40,86]. Notably, the interest in collagen as a biomaterial depends upon its source and diverse morphologies [28].

Moreover, collagen has the ability of producing sheets, tubes, powders, fleeces, injectable solutions, and dispersions, which expand its usage in the medical sphere. These applications of collagen are tested in drug delivery systems in ophthalmology, wound and burn dressing, tumor treatment, and tissue engineering [41]. PSC from sea cucumber (*Stichopus japonicus*) has been investigated for its ability in wound-healing [96] and has shown increased cell migration and proliferation as well as wound-healing effects in human keratinocyte cell lines compared to conventional collagen [96]. These findings demonstrate the potential of sea cucumber collagen for use as an alternative collagen source in biomedical applications.

The collagen as a biomaterial can be used under different fields of applications such as tissue engineering, bone substitutes, eye implants, drug delivery matrix, gene delivery matrix, protein delivery matrix, and as a useful biomaterial which forms organoids or neo-organs in gene therapy [145]. Furthermore, the use of collagen in cosmetic surgeries is one of its significant applications due to commercial influence in the industrial sphere related to biocompatibility and safety [86]. Besides, collagen is often used as a hemostatic agent, and surgical suture ascribed to its shorter period of healing time over other traditional methods [41,86,145]. Furthermore, the use of marine collagen is becoming popular in the field of tissue engineering [146]. Carvalho et al. [147] studied the marine-derived type I collagen with receptors at the cell surface and its potential of involving cell adhesion, differentiation and growth and developed novel biomaterials using combination of other biopolymers with collagen. A recent study on Jellyfish collagen as biomaterial also demonstrated its potential as a possible alternative to type I collagen source in fibrillar or nonfibrillar form for tissue engineering studies and industrial use [148].

Echinoderm originated mutable collagenous tissues have the potential to develop the collagen barrier-membranes for tissue regeneration applications [146]. The research on different echinoderm models including sea cucumber (*Holothuria tubulosa*), sea urchin (*Paracentrotus lividus*), and starfish (*Echinaster sepositus*) substrates were used to determine their compatibility to exploit as collagen barrier membranes for guided tissue regeneration (GTR) process and demonstrated similar cell morphology of all tested materials to commercially used bovine collagen substrate and echinoderm collagenous tissues [146]. Another specific advantage of echinoderm derived collagen is the tendency to maintain its original structure even after the extraction process [149]. The scaffolds, central fabrication to tissue engineering technology made of soluble jellyfish or squid collagen, have shown lower immunogenicity and higher cell viability compared to other biomaterials like bovine collagen [149,150]. Furthermore, collagen and its hydrolysates are used as a supplement for bone integrity, brittle nails treatment, and osteoarthritis pain [147,148,149,150].

In addition to the advantages linked to sea cucumber derived collagen, most Asians are still considering sea cucumber as a traditional medicine for treating asthma, hypertension, rheumatism, and anemia [151]. Hence, application of collagen in the biomedical sector has expanded to the field of pharmaceutical industries as well as in tissue engineering as injectable matrices, scaffolds for bone reconstruction, vascular, and cardiac reconstruction [19,20].

However, limitations of using marine collagen as biomaterial are inevitable. The diversities of cross-link density, fiber size, and trace impurities are factors that hinder the use of isolated collagen. In addition, variability in enzymatic degradation rate and nature of hydrophilicity, production yield over mammalian collagen and high cost associated with the preparation of type I collagen are considered as some major drawbacks for the use of marine collagen [10,14,15,19,20]. Specifically, further in vitro and in vivo studies are necessary to extensively investigate the biocompatibility and immunogenicity of marine-derived collagen, including those from sea cucumbers for human clinical applications [146].

### 5.2. Non-Biomedical Applications

The industrial use of collagen in classical food, photographic, cosmetic and many other applications (involvement of leather production, producing gelatin-like hydrolysates) are mainly based on its unique functional and technological properties [9]. The use of collagen as a source of glue has an 8000 year history in protection of embroidered fabrics and tools and 4000 years as an adhesive used by Egyptians [8]

More recently, collagen is used widely in new food product development as a clinically proven healthy nutritious food supplement. Collagen supplements are considered as an anti-aging agent which are capable of upholding skin, hair, nails, and body tissues [71]. Moreover, food products, including gelatin-like collagen hydrolysates, are utilized in confections, low-fat spreads, baked, and meat products [8].

Furthermore, collagen is also popular as a food additive due to its ability to improve rheological properties of meat products and act as an emulsifier in acidic products. de Castro et al. [152] reported that heat-treated collagen has a high potential of use as emulsifier. Heating under acidic condition leads to reducing the charge of protein and as a consequence increasing its solubility which exerts a positive influence on the emulsion ability.

Edible films are widely used in the food industry as the barrier for moisture and oxygen to improve the shelf life of food [71]. Food grade collagens are widely used as sausage casings and these casings could be developed using regenerated bovine hides [153]. Other than sausages, edible collagen films and coatings are also used on different meat and fish products such as hamburgers, netted roasts, boneless hams, and fish fillets [153]. The application of collagen films and coatings increases juiciness and reduces cook shrinkage in most of these foods. Moreover, the potential of using these coatings as a protective barrier and replacing plastic wrappings has been shown to control oxidation, color, microbial growth, and to retain sensory attributes of meat products [71,153]. Besides, these films and coatings could be utilized as carriers of bioactives, including antioxidants, antimicrobials, colorants, and flavorants [153].

Furthermore, collagen has the potential to be used as biobased food packaging material [154]. In addition, hydrolyzed collagen may be used as a fat replacer in processed meat products like sausages. Ibrahim et al. [155] used fish collagen hydrolysates as fat replacer in the production of buffalo patties and reported that inclusion of hydrolyzed collagen afforded high protein, low-fat, and better textural characteristics compared to the buffalo patties without hydrolyzed collagen [156].

The recent development of inclusion of collagen in beverages has gained the interest of the global food market. Collagen from soy, cocoa, and cappuccino, juice with collagen and birds nest drink ware examples of some collagen-based drinks [71]. The triple helix and rod-like structure of collagen can be used for clarification of cloudy alcoholic beverages by aggregation of the yeast and other insoluble particles [144]. Furthermore, Bilek, and Bayram [157] indicated the successful addition of hydrolyzed collagen to beverages for enhancing their nutritional and functional properties which is now widely used as a food ingredient in functional foods [157]. The addition of hydrolyzed collagen enhances the nutritional and functional properties of orange juice and the physicochemical and microbial properties of fermented dairy products [156].

However, collagen-infused liquid is generally manufactured for cosmetic purposes such as improvement of moisture-retaining properties of the skin and prevention of forming wrinkles [157]. Therefore, collagen has now gained much attention as an emerging source for cosmetic products. The cosmetic industry uses collagen as a treatment for skin replacement and other beauty-related products due to its close relationship with skin aging and abundance in the form of connective tissue in the human body, especially in skin and bones [158]. A recent study on extraction and characterization of collagen from sea cucumber (*Holothuria cinerascens*) revealed the high moisture retention and moisture absorption capacity compared to collagen extracted from tilapia and porcine skin [83]. Besides, PSC from *Holothuria cinerascens* were found to be rich in polar groups, including carboxyl and hydroxyl groups and capable of forming hydrogen bonds with water. This unique characteristic allows collagen to interact with water hence allows its use in moisturizers [83,158].

Kim et al. [159] also studied the skin whitening and wrinkle improvement efficacy of the glycoprotein fractions from liquid extracts of boiled sea cucumber and found that glycoprotein higher than 50 kDa fractions had the potential for use as a cosmetic ingredient. Type I collagen is the most abundant collagen type produced by skin fibroblasts. Numerous studies have proven that collagen derived from sea cucumbers also represents the type I collagen group. Kupper et al. [160] investigated the application of collagen/hyaluronic acid-based microemulsions from sea cucumber *Holothuria cinerascens* as the transdermal carrier with the focus on anti-aging research products including anti-wrinkle creams. At this point, collagen derived from cold-water fish skin, including cod, haddock, and salmon are being widely used in the cosmetic industry [161,162].

## 6. Pre-Treatment, Extraction, Isolation, and Purification

Collagen exists in the insoluble macromolecular structure of the body. Therefore, procedures for the preparation of collagen consist of several key steps including pre-treatment, extraction, separation, purification, and characterization [83,162,163,164]. The critical factor in collagen extraction is the removal of covalent intra- and intermolecular cross-links [162]. Collagen extraction procedure includes two steps of pre-treatment of raw material and then extraction of collagen. Preparation procedures can vary based on the type of raw material. However, general steps, including cleaning, size reduction and pre-treatment procedures, are essential steps before extraction in order to prevent contamination. The removal of impurities may also assist in maximizing the yield and quality of the extracted collagen [10]. Table 5 summarizes the pre-treatments and extraction methods used in collagen extraction from different sea cucumber species.

### 6.1. Pre-Treatment

Both acidic and alkali pre-treatments are widely used in collagen extraction procedures [28]. Mild chemical treatment is generally used prior to extraction, mainly due to the cross-linked nature of collagen [162]. For instance, acidic pre-treatment procedure is favorable for the extraction of collagen from raw materials with fewer cross-links, as acidic solution helps to break non-covalent bonds under controlled temperature [57,162]. In contrast, in alkaline pre-treatment procedures, the basic solution removes non-collagenous proteins, lipids, pigments, and calcium as well as other inorganic material [10]. Factors such as time, temperature, and concentration of the solution play essential roles for effective removal of these non-collagenous materials during alkaline pre-treatment [161]. According to Schmidt et al. [162], the concentration range of 0.05–0.10 M of NaOH can be considered as being adequate for pre-treatment. Moreover, the same concentration range protects the acid soluble collagen and structural modifications at different temperatures from 4 to 20 °C. In contrast, 0.5 M NaOH causes structural modification at 15 and 20 °C while 0.2 and 0.5 M both can lead to the loss of acid-soluble collagen. In addition, alkaline method is also practiced in treating thick hard raw material which requires effective penetration through raw material to cleave the inter- and intramolecular cross-links of collagen (Table 4) [162,166]. Furthermore, alcohol is effective for the removal of fat and pigments from seafood and butyl alcohol is a widely used alcohol, among others [10,161].

However, most of the pre-treatments in collagen extraction from sea cucumber include ethylenediaminetetraacetic acid (EDTA) for the demineralization process [10,166]. The chelating action of EDTA for calcium ion facilitates the collagen extraction process by using the substrate to a greater extent [161,162].

### 6.2. Extraction Methods

**Figure 7:**
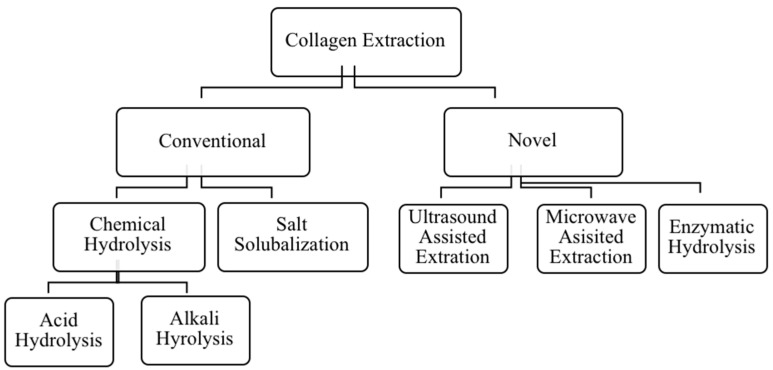
Collagen Extraction Methods.

Collagen extraction methods can be divided into two main groups as conventional and novel. According to the extraction process, both conventional and novel methods can further be classified into several types such as chemical hydrolysis, enzymatic hydrolysis, ultrasound-assisted extraction, and pressurized liquid extraction (Figure 7). The yield and properties of collagen depend on the extraction method employed. Most of the extraction processes are carried out under controlled temperature (4 °C) to prevent collagen degradation [10]. Furthermore, functional properties of the extracted collagen, including the length of polypeptide chains and viscosity, solubility, water retention, emulsifying, are also affected by the extraction method. In addition, the variability of processing parameters, pre-treatment methods, storage conditions, and the nature of raw materials also influence the quality of extracted collagen [8].

#### 6.2.1. Conventional Methods

Conventional collagen extraction methods mainly include chemical hydrolysis and salt solubilization. Acid and alkali solubilization extraction methods have been used for crude collagen extraction and come under the chemical hydrolysis category. The chemical hydrolysis method is widely used over the salt solubilization method for industrial collagen production [10,162].

##### Salt Solubilization

When it comes to the extraction, neutral saline solutions are used due to the solubility of collagen in salt. Sodium chloride, phosphates, citrates, or Tris-HCl are mostly used neutral saline solutions [162]. Collagen extraction using NaCl solution is referred to as salt-solubilized collagen. The salt solubilization extraction method is used for collagen extraction from different tissues, including bones, cartilages, skin, and scales. However, the properties of extracted collagen are based on the salting-out method of the salt solubilization extraction procedure [167]. Generally, solubilization extractions are followed by either acid or enzyme assisted extraction [168]. Ran and Wang [169] revealed the low efficiency of using salt solubilization for collagen extraction. Moreover, it is mandatory to control the concentration of salt due to the nature of collagen molecules. Salt concentration < 1.0 mol L^−1^ is used for dissolution of type I collagen, while concentration > 1.0 mol L^−1^ is best for the precipitation of type I collagen [163]. Therefore, salt or saline solution extraction has more limitations compared to chemical hydrolysis processes.

##### Chemical Hydrolysis

The chemical hydrolysis method is mainly categorized into acid and alkali hydrolysis. The acid hydrolysis method is extensively used and both organic and inorganic acids are able to cleave the bonds between collagen molecules and improve the extraction of collagen fibrils. Under acidic conditions, collagen molecules get more positively charged [163] and this positive charge facilitates their solubilization by creating the repulsion among tropocollagen molecules [10] Organic acids, including acetic, citric, lactic, and chloroacetic acid and inorganic acids such as hydrochloric acid, are used for the isolation of collagen [162,166,170]. However, organic acids are more effective compared to inorganic acids in cleaving the crosslinks of collagen molecules and result in higher extractability of collagen [170,171]. Acetic acid is the most commonly used organic acid which change the electrostatic nature of collagen to enhance its solubility and extractability [10,170,171].

Generally, the acid hydrolysis procedure uses 0.5 M acetic acid and the reaction mixture is continuously stirred for 24–72 h [169]. In order to obtain the crude collagen powder, sequential filtration, precipitation with NaCl and centrifugation are conducted. The filtrate should then be dissolved in acetic acid (0.5 M) followed by dialysis using 0.1 M acetic acid for two days and subsequently distilled water for two days [162].

Some extraction requirements are varied depending on the type of raw material. For example, extraction procedure for collagen from marine sources may need to be maintained at 4 °C with constant stirring for 24–48 h. The resultant extraction fraction can also be varied according to the concentration and proportion of the acid used [10,21]. de Moraes and Cunha [172] revealed that the collagen extracted under acidic pH and high temperature possessed low molar mass and the hydrolysates formed firmer gels [162]. Thus, the pH of the extraction medium may influence the nature and the physicochemical properties of extracted collagen. In addition, a positive relationship was reported between extraction time and the yield of the extracted collagen [170]. However, Benjakul et al. [166] suggested that sequential extraction cycles can give a higher yield of acid soluble collagen instead of extending the extraction time. Temperature is considered as another important variable which can directly influence the yield of collagen. Acid soluble collagen extraction can be performed within the temperature range of 4–20 °C without harming the nature of the collagen [171]. According to Pal et al. [10], acid hydrolysis process can also be conducted using 6M hydrochloric acid under high-temperature range from 110 to 120 °C for a longer period (18–48 h) with the resultant collagen being similar to that obtained under general conditions.

However, Pal et al. [10] stated that the yield of collagen could vary according to the nature of raw material and other variables related to the extraction process. Factors including type and source of raw material (species, age), extraction process, concentration and proportions of acid, extraction temperature, pH, and process time affect the yield of crude collagen. In alkali hydrolysis, strong alkali solutions are used for the dissolution and degradation of collagen. The two commonly used alkali solutions are sodium and potassium hydroxide [10]. In addition, calcium oxide, calcium hydroxide, and sodium carbonate are also used as extractants [173]. Moreover, alkali has strong hydrolysis ability and may hydrolyze proteins by acting on collagen fibrils [163,173]. However, amino acids like serine, cysteine, histidine, and threonine may be destroyed due to extreme extraction conditions [10,173].

#### 6.2.2. Novel Methods

There are several novel methodologies for collagen extraction which address the limitations of conventional methods. Mainly, enzymatic hydrolysis is a technique that belongs to the realm of green chemistry. Furthermore, a combination of multiple methods or hybridization of chemical and enzymatic hydrolysis may be used for maximizing the yield of collagen extraction and increasing the purity of extracted collagen. The acid-enzyme, alkali-enzyme, and acid-alkali combined hydrolysis methods have been studied for their applicability at industrial levels [163].

Different novel approaches have been investigated in collagen extraction to find the most cost-effective procedure with minimum environmental impact. Ultrasonic [174,175,176,177] supercritical fluid, microwave [127,178], and high-pressure extraction are under investigation in terms of industrial applications [10]. Most of these methods need extreme conditions such as high heat and pressure. Thus, the denaturation of extracted protein might occur. However, a significant number of studies based on sea cucumber collagen and collagen hydrolysates have focused on enzymatic hydrolysis [99,102,120,127,165].

##### Enzymatic Hydrolysis

Employing enzymes for the extraction of collagen is widely used and regarded as one of the convenient biological methods for industrial application [162]. The enzymatic extraction process has been developed to maximize the collagen yield as it has high reaction selectivity and less destructive effect on molecular structure of collagen [163]. Moreover, enzymatic hydrolysis is an efficient procedure as it possesses more favorable characteristics over the chemical hydrolysis method. Despite the higher cost, enzymatic hydrolysis method has significant advantages compared to chemical hydrolysis methods such as high specificity, controlled degree of hydrolysis, moderate reaction conditions, final hydrolysate with least salt content, lower waste production, and a higher yield of collagen.

Various proteolytic enzymes from animal origin (trypsin, pepsin), plant sources (bromelain, papain, ficin) or commercial proteolytic enzymes (collagenase, proteinase K, Alcalase, Nutrase, Flavourzyme, Protamex) have been used for the enzymatic hydrolysis process. Among these, pepsin from animal origin is the most extensively used enzyme [10,166,171]. As the widely used enzyme, pepsin has the ability to cleave the non-helix peptide chain of collagen protein right at the 3/4 position of N-terminal, so the helix peptide chains of collagen remain unchanged [163]. Studies on sea cucumber collagen have shown that pepsin solubilization extraction has no effect on its triple helix structure [14,98,99,102,142,165]. Sea cucumber collagen was extracted by hydrolyzing non-helical telopeptides in cross-links using pepsin without degenerating the integrity of the triple helix [120]. During acid hydrolysis, salt links and Schiff base in cross-links are degenerated with weak acid. Thus, PSC has a high rate of extraction compared to acid soluble collagen. The extraction efficiency of acid solubilized collagen (ASC) from *Parastichopus californicus* was lower compared to PSC [120]. Zhong et al. [101] also reported a higher PSC compared to ASC from *Stichopus monotuberculatus* and indicated the predominant impact of covalent cross-links in the telopeptide region of the peptides on collagen solubility.

Moreover, papain has also been reported to control the cleavage of the substrate protein. Jin et al. [127] used papain with microwave radiation to extract collagen from sea cucumber (*Acaudina molpadioides*) and reported that papain can be used to induce collagen extraction from sea cucumber body wall. However, pepsin was found as one of the best enzymes which could maintain the degree of cleavage of the substrate protein [162]. Pepsin soluble collagen has high purity compared to other extracted collagens, mainly due to its ability to hydrolyze non-collagenous proteins. Most of the other non-collagenous materials can be removed from the collagen by salt precipitation and dialysis. Besides, pepsin can also increase the extraction efficiency of collagen by improving its solubility in acid solution. In addition, the degree of cross-linking at the telopeptide region of the peptides determines the yield of pepsin soluble collagen. Adibzadeh et al. [98] reported a lower yield of pepsin soluble collagen isolated from *Holothuria parva* than *Parastichopus californicus* and *Stichopus monotuberculatus* due to the higher degree of cross-links in *Holothuria parva* compared to *Parastichopus californicus* and *Stichopus monotuberculatus*.

In most research efforts, exogenous enzymes are often used for the extraction process due to their ability to control the hydrolysis with a comparatively lower processing time than other methods. However, numerous studies have been conducted to investigate the effect of endogenous enzymes of sea cucumber species on collagen fibrils. Endogenous enzymes including cysteine proteinases [141] serine proteinases [179] and matrix metalloproteinases [97,101,118] have been characterized from various sea cucumber species and are involved in the autolysis of sea cucumber. Yan et al. [179] demonstrated that serine proteinases from sea cucumber could have the ability to cleave the collagen cross-links. Similar findings were revealed using trypsin-assisted (type of serine proteases) degradation of collagen fibrils isolated from *Stichopus japonicus* [118]. Nevertheless, cysteine and serine proteases partially hydrolyze the surface of collagen fibrils [102,118]. Besides, metalloproteinases have also been investigated to examine their activity on sea cucumber collagen. Liu et al. [119] revealed that collagenase type I, which belongs to the metalloproteinases, was involved in the unfolding of collagen fibrils by degenerating monomeric collagen.

##### Ultrasound-Assisted Extraction

Ultrasound technique is used for the extraction of collagen as an alternative to conventional methods in order to reduce processing time and improve the extraction yield [74]. Ultrasound is a high frequency wave (20 kHz) which exceeds the hearing capacity of humans (16 kHz) and uses the energy of sound waves to transfer mass by a wet process [162,180]. Energy generated by ultrasonic waves affects the kinetic energy of the particles in the treated substance and the phenomenon is known as sonication. Moreover, the effect of ultrasound in a liquid system or the cavitation is induced by vibration [175]. The principal mechanism of ultrasound is generating bubble cavitation in the biological matrix [180]. Therefore, during the process of sonication, ultrasound generates cavitation bubbles and by resulting high temperature and pressure, these bubbles collapse [162]. Kim et al. [175] extracted collagen from sea bass skin using ultrasound-assisted extraction and reported no alterations in the basic structure of the resultant collagen. Furthermore, the yield of collagen was based on the amplitudes and duration of the treatment, as a higher yield was reported with higher amplitudes and short time duration. However, they recommended further studies to verify the influence of the process on structural damages to the extracted collagen. Recently, Song et al. [181] focused on developing an industrial ultrasound system for mass production of collagen from fish skin and reported a two-folds higher collagen yield using ultrasound-assisted extraction compared to the conventional acid-assisted extraction.

Ran and Wang [169] investigated the effect of combination of pepsin and ultrasound-assisted extraction to obtain bovine tendon derived collagen and reported a higher efficiency of extraction as well as a better quality of extracted collagen. Li et al. [182] also indicated that the combination could increase the yield and reduce the required time. As a newly emerging technique, ultrasound-assisted extraction has several advantages over the conventional extraction methods, including no complex procedures, environmentally friendly, safe to practice, short processing time, and economic viability. However, to date, no in-depth research has been conducted on identifying the effect of ultrasound on sea cucumber collagen. Thus, more research is needed for a thorough investigation on the quality of extracted collagen as well as to overcome limitations such as controlling amplitude with the distance and inhibition of enzyme activity [10,162,182].

##### Microwave-Assisted Extraction and Other Methods

The microwave-assisted extraction process is based on the electromagnetic waves and the disruption of the cell structure [127,183]. Microwave radiation can penetrate the interior of proteins and facilitate the extraction by loosening their structures from the cell matrix [127]. Microwave-assisted extraction of collagen is often followed by enzyme hydrolysis as acid or enzyme-assisted hydrolysis can be enhanced by using microwave power in order to complete the hydrolysis of collagen [183]. Jin et al. [127] investigated the microwave-assisted enzymatic hydrolysis of collagen from sea cucumber *Acaudina molpadioides* and reported significant bioactivities of produced peptides from collagen fibrils.

Besides the microwave treatment, another recent method, high-pressure solvent extraction, was reported for extracting collagen hydrolysates [10]. The high-pressure liquid extraction technique operates at temperature and pressure within the range of 50–250 °C and 3.5–20 MPa, respectively [184]. As high-pressure solvent exceeds its boiling temperature in most cases, water is used as an alternative extraction solvent. Therefore, the method is usually referred to as pressurized hot water extraction or subcritical water extraction [185]. Gomez-Gullien et al. [186] investigated the extraction of gelatin from fish skins using high-pressure treatment and reported significantly shorter extraction time and superior quality gelatin compared to other conventional methods. In addition, studies on pacific blue whiting [187], using high-pressure treatment (300–400 MPa, reported no significant effect on extracted collagen. However, further studies are needed to confirm the quality and functionality of the resultant collagen or collagen hydrolysates extracted using pressurized liquid extraction method.

### 6.3. Isolation Methods

**Figure 8:**
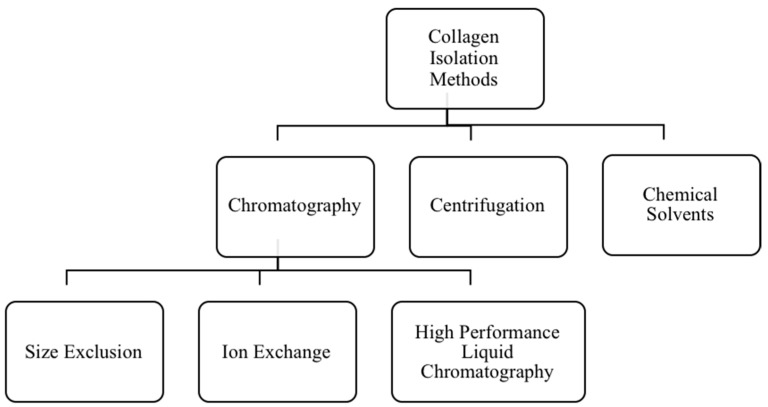
Collagen isolation methods.

Developing a standard isolation method for collagen becomes a difficult task mainly due to the extreme diversity of tissues and existence of genetically distinct types of collagen. Moreover, the relationship between intermolecular interactions and collagen solubility in the solvents used are essential prior to selecting a method for isolation [93]. There are various isolation methods based on chromatographic methods (including size exclusion, high-performance liquid, and ion-exchange chromatography, etc.), centrifugation, and solvent extraction (Figure 8).

#### 6.3.1. Chromatography

Chromatography is a proven technique for separating and analyzing the components of a complex mixture, and it is most effective when the mixture is a biological extract [150,188,189]. Chromatographic separation is always linked with the migration of the components through the column [150]. Based on principles such as adsorption, partition, ion-exchange, or molecular exclusion, chromatographic procedures are used for separation purposes.

As an effective method of protein isolation, chromatographic columns are mostly used after a centrifugation or filtration process. When considering the columns, a wide range of chromatographic column packing materials are available at commercial level including gel filtration medium, ion exchange, reversed-phase packing, hydrophobic interaction adsorbent, and affinity chromatography adsorbent [189,190].

Among the chromatographic methods, size exclusion chromatography (SEC) is extensively used analytical technique for quantitative and qualitative analysis of biological extracts and often used to determine the molecular weight and molecular weight distribution [190]. Moreover, application of buffer exchange procedures, studies related to interaction and concentration of solutes, solute diffusivity and shape determination are considered for selecting the purification and fractionation methods for protein aggregates [189,190].

Gel filtration is one of the widely used techniques in SEC. Cui et al. [16] carried out gel filtration chromatography to purify the extracted collagen from *Stichopus japonicus*. Besides, a recent study on isolation of bioactive peptides from collagen hydrolysates from sea cucumber *Acaudina molpadioides* also used gel filtration chromatography to separate the peptides based on their molecular size [127]. The authors used SEC to characterize the antioxidant peptides from microwave-assisted hydrolysates of sea cucumber collagen and performed further analysis to determine the peptide sequences.

Numerous studies have been conducted by applying the SEC method mainly because it is mild and has minimal impact on the conformational structures of the molecules [190]. Furthermore, SEC has several favorable characteristics compared to other analytical separation methods, including its high recovery rate and compatibility with a range of physiological conditions. Hence, these features expand the applicability of SEC in industrial level purification procedures [188,189,190].

Ion exchange chromatography (IEC) related to collagen identifications has been used to characterize the crosslinks present in different collagen types. Naffa et al. [191] characterized the collagen type I cross linked from bovine skin and used IEC as one of the isolation methods for separating the diastereoisomers of hydroxylysinonorleucine. In general, IEC is a non-denaturing technique for analyzing and characterizing charge variants of protein samples [192]. Among the different IEC methods, cation exchange chromatography is the most efficient chromatographic method for purification and characterization of protein. Cation exchange columns were used to measure the collagen crosslinks present in tissue samples [193]. These IEC methods were employed to characterize both intact and digest forms of proteins, including collagen [192,193].

High-Performance Liquid Chromatography (HPLC) is one of the most robust and efficient chromatographic techniques. Reversed-phase-HPLC (RP-HPLC) is used in the characterization and purification process in collagen peptides [10]. The RP-HPLC method is often employed for the separation of low-molecular-weight peptides and for amino acid analysis. Dong et al. [105] investigated the molecular weight distribution of collagen peptides isolated from *Apostichopus japonicus*. Zhu et al. [95] further purified the pepsin soluble collagen isolated from *Stichopus japonicus* by removing carbohydrate moieties from the collagen fibrils and used a multi-step gradient elution system coupled with a UV-visible spectroscopy to detect the collagen and carbohydrate peaks separately. In addition, RP-HPLC was employed to determine the amino acid sequence of the purified fractions collected from SEC or IEC. Jin et al. [127] analyzed the fractions of collagen hydrolysates from *Acaudina molpadioides* and determined the amino acid sequence of bioactive peptides separated from the SEC.

Furthermore, liquid chromatography and mass spectrometry, including matrix-assisted laser desorption/ionization time-of-flight (MALDI-TOF) methods are often combined with HPLC. The primary objective of the association of sophisticated techniques with the HPLC method is to advance the identification of collagen and collagen peptides [190,191,192,193].

#### 6.3.2. Centrifugation

Centrifugation is routinely used for the purpose of recovering precipitates, especially in the protein purification process. In addition, density gradient centrifugation and fractionation of subcellular particles and nucleic acid are the common applications of centrifugation for separation of two immiscible liquid phases [150,162]. During centrifugation, time, velocity, and other geometrical factors related to the rotor are dependent on the method and the type of sample [162,189,190]. In clarification procedures, refrigerated high-speed centrifugation is commonly used for any cell homogenate [150]. Centrifugation is more convenient in a laboratory scale compared to filtration. Most of the time, the standard centrifuge temperature is around 0 °C or below. The centrifugation step is often considered as essential in most of the purification methods.

#### 6.3.3. Use of Non-Aqueous Solvents for Isolation and Purification

There are many specialized methods for protein extraction which can be used directly for chromatographic separation either after centrifugation or filtration [193,194]. Extraction yield and properties of the resultant compound are directly linked to the extraction method [10]. The composition of suitable extraction medium needs to be considered, including pH, buffer salts, detergents, reducing agents, proteolytic inhibitors, and bacteriostatics. Mocan et al. [194] stated that developing a standard method for isolation of all types of collagen from different tissues is a difficult task due to the extreme diversity of both the tissue and collagen type.

### 6.4. Assaying of Isolated Collagen

#### 6.4.1. Western Blotting

For collagen assay, western blot technique is used among southern and northern blot which are generally employed for DNA and RNA assays, respectively. The western blotting technique is used to separate and identify proteins [195]. The phenomenon behind this blotting technique is to transfer electrophoretically separated macromolecules from a gel to a blotting medium. In blotting or immobilizing medium, electrophoresis pattern can be observed which allows subsequent reaction between separated macromolecules and probes [195,196]. In other words, through gel electrophoresis, a protein mixture is separated according to the molecular weight and type of its components, and then transferred to the blotting medium to produce a band for each protein in order to detect the type of protein utilizing a specific affinity of protein [195]. Thus, three major steps are involved in the western blotting technique to identify proteins; these include (a) separation based on molecular size; (b) transfer to immobilizing medium; and (c) marking target protein using a specific or labeled antibody.

Procollagen and collagen can be identified using the western blot technique [196]. Quiñones et al. [197] used immunoblotting to study the regenerative capacity of internal organs of sea cucumber *Holothuria glaberrima* and western blotting results confirmed the decrease in fibrous collagen content during regeneration.

#### 6.4.2. SDS-PAGE

Sodium dodecyl sulphate polyacrylamide gel electrophoresis (SDS-PAGE) can resolve the individual components of a complex protein mixture and it is the most widely used laboratory technique for protein identification [188,198]. This technique is often used for fractionation and quantification of proteins in connection with either mass spectrometric identification or immunological test [199]. Even though SDS-PAGE method is used as a tool to characterize proteins according to their size, charge, relative hydrophobicity and abundance with the newly emerging techniques such as protein sequencing, amino acid compositional analysis, peptide profiling hinders the use of SDS-PAGE for analytical purposes [27,199]. Table 6 summarizes the studies conducted using different sea cucumber species to determine the subunit composition of isolated collagen. This technique is one of the highly efficient methods of protein recovery, but there are few limitations associated with it including (a) relatively slow isolation rate (b) possible contamination with impurities (sodium dodecyl sulphate (SDS), salts, etc.), (c) possibility of damaging the peptide chain during elution or staining and occurrence of chemical modifications, and (d) resulting in N-terminal blockage [198,199].

#### 6.4.3. Spectrophotometric Analysis

Purified proteins may be analyzed by ultraviolet absorbance or fluorescent spectroscopy [200]. A wavelength of 200–400 nm is the most appropriate range to analyze the collagen derived from marine sources [10]. However, the maximum absorbance wavelength of collagen should include the range of 210–230 nm due to the content of tyrosine, tryptophan, and phenylalanine present in collagen [16,101].

Zhu et al. [95] used UV absorbance spectroscopy at 220 and 233 nm to investigate and characterize purified PSC content of sea cucumber *Stichopus japonicus*, respectively. Abedin et al. [99] used the same technique for characterization of collagen extracted from sea cucumber *Stichopus vastus* and observed a single maximum peak at 215 nm corresponding to the UV–VIS spectrum of type I collagen.

New analytical techniques with different mass spectrometric approaches have been introduced for protein analysis [200]. Mass spectrometry is a sensitive technique for detection, identification, and quantification of molecules based on mass to charge ratio of their ions. It provides novel means to analyze collagen cross-links [200,201]. Samples for mass spectroscopy can come directly from SDS-PAGE or using different protein purification methods, including chromatography. Moreover, adequate peptide solubilization before loading is one of the vital steps in sample preparation for mass spectrometric analysis [201].

Fourier transform infrared (FTIR) spectroscopy is another popular technique for analyzing the structure of proteins, especially characterization of their secondary structure. In collagen characterization studies, FTIR spectroscopy plays a vital role as it allows confirmation by absorption wavenumber of each amide band [105].

FTIR spectra of extracted collagens, especially from seafood by-products, indicates unique peaks of amide bands and provide evidence of the triple helical structure of collagen [10] indicating the direct relationship of amide bands and configuration of the polypeptide [202]. Generally, amide A band (3400–3440 cm^−1^) is related to N-H stretching vibration, amide I band is associated with stretching vibration of the carbonyl groups along the peptide backbone while amide II is associated with the N-H deformation and amide III is due to C-N stretching and N-H deformation [95,99,202]. Among the amide bands, amide I band is considered as being a crucial factor in determining the secondary structure of protein molecules. Analysis of the amide I band in infrared (IR) spectra indicates the characteristic structural changes of triple helix in the collagen molecule that are stabilized by hydrogen bonds present in C=O and adjacent groups [105]. Furthermore, the triple helical structure of collagen is confirmed from the absorption ratio between 1236.5 and 1449.5 cm^−1^ of amide III band, which is approximately equal to 1.0 [95,99]. Lower structural stability of collagen correlates with the higher wavenumber of amide bands [105].

Abedin et al. [99] and Zhu et al. [95] used FTIR method for pepsin soluble collagen derived from sea cucumber species and observed the absorption bands of amide I, amide II, amide III within the range 1600–1700, 1550–1600, and 1220–1320 cm^−1^, respectively. A recent study on thermostability of sea cucumber *Apostichopus japonicas* used FTIR spectra to evaluate the secondary structural deformation of collagen during thermal treatment [105]. Thus, FTIR analysis of natural or synthetic collagen has been widely used to elucidate structural characteristics of collagen.

### 6.5. Characterization of Isolated Collagen

#### 6.5.1. Differential Scanning Calorimetry

Thermal denaturation of collagen is a sequential and irreversible process related to the unfolding of its unique triple helix structure [105]. The relationship between protein denaturation and thermal activity is monitored using differential scanning calorimeter (DSC). Thermogram produced by the DSC helps in the identification of the nature of target protein under thermal stress. Midpoint or the lowest point of the endothermic peak in the thermogram indicates the maximum transition temperature, Tm [203], whereas Td refers to the denaturation temperature.

The helical structure of collagen is denatured and completely breaks down at 8 and 45 °C, respectively [202]. The thermal denaturation temperature of the collagen solution is the temperature at which 50% of the change in viscosity occurs. Fraction change is calculated using Equation (1).

Fraction change = [(ε2/C) − (ε3/C)]/[(ε1/C) − (ε3/C)](1)

where C = collagen concentration (mg/mL), ε1 = specific viscosity at 8 °C, ε2 = specific viscosity at measured temperature (°C), and ε3 = specific viscosity at 45 °C.

Hence, denaturation temperature is based on the changes in viscosity. The thermal determination curve is obtained by plotting fractional viscosities against temperature. The denaturation temperature can be observed where the fractional viscosity is predicted to be 0.5 [64]. Furthermore, thermal depolymerization occurs with increasing temperature, which leads to disruption of the triple helical structure by breaking the hydrogen bonds [99]. The unwinding of the triple helix structure results in the denaturation of secondary or tertiary structures of collagen, but the primary structure remains intact. Liu et al. [103] observed similar results and depicted that thermal denaturation of sea cucumber collagen is a time dependent-irreversible transformation of the native helical structure. The fractional change of PSC from the integument of sea cucumber was decreased with increasing temperature and thermal stability of collagen was correlated with the environmental and body temperature of the organism [99]. According to most reported results, the thermostability of triple helical structure of sea cucumber derived collagen is lower compared to mammalian collagen [120].

#### 6.5.2. Tyrosine Measurement

Tyrosine content can be used for determining the collagen content of a sample [64]. Collagen may be hydrolyzed at 105 °C in 6 M hydrochloric acid for 24 h under a nitrogen atmosphere and amino acids then quantified using liquid phase ion-exchange chromatography [196]. In contrast, Lin and Liu [48] measured the tyrosine content using near UV absorption spectrum (chromophores of tyrosine). In order to analyze the purified extracted collagen, tyrosine measurement is widely used as it shows the integrity of non-helical telopeptides and other protein contaminants [48].

#### 6.5.3. Hydroxyproline Determination

Colorimetric assay of hydroxyproline is a robust and reliable method for analysis of collagen purity. Collagen is rich in hydroxyproline that can be differentiated from the negligible amount present in other proteins [204]. Moreover, hydroxyproline plays a vital role in thermal stabilization of collagen as it forms hydrogen bonds between collagen peptides. Thus, the content of hydroxyproline has a direct relationship with the thermal stability of collagen [161].

Collagen is hydrolyzed at 105 °C in 3.5 M sulfuric acid for 16 h to determine the hydroxyproline content. The colorimetric method is performed and the hydroxyproline content is then converted to total collagen using a factor of 7.57. The determination of collagen is usually conducted using international organization for standardization (ISO) 3496: 1994 standard method for meat and meat products. The final value is expressed in terms of the ratio of extracted hydroxyproline compared to its initial concentration in the source material [161,205].

## 7. Functional Properties of Collagen

Interest in functional properties of collagen extracted from different sources, including animal, marine organism, and industrial by-products, has been increasing during the past few decades. According to Gomez-Guillen et al. [9], functional properties of collagen and gelatin can be divided into two main categories as properties associated with gelling behavior, and surface behavior. Properties associated with gelling behavior include (a) gel formation, (b) texturing, (c) thickening, and (d) water-binding capacity while properties related to their surface behavior include (a) emulsification, (b) foaming and stabilization, (c) adhesion and cohesion, (d) colloid function, and (e) film formation [9,150,171].

### 7.1. Gelling and Hydrophilic Properties

The process of collagen gelation is the aggregation of collagen molecules that can be achieved by heating either in acid or alkali [8] and induced by alterations of processing parameters such as ionic strength, pH, and temperature. During thermal solubilization of collagen, a considerable amount of intra- and intermolecular cross-links are cleaved. The aqueous solution of gelatin and collagen possesses the ability to swell by covalently linking with matrices [9]. Liu et al. [120] and Abedin et al. [99] evaluated and compared the gel-forming ability of sea cucumbers *Parastichopus californicus* and *Stichopus vastus* derived collagen with calfskin collagen. The findings of these studies revealed that ionic strength and pH were the predominant factors determining the gel-forming ability of collagen isolated from sea cucumbers. Moreover, calfskin collagen exhibited higher gel-forming ability compared to sea cucumber-derived collagen. The difference might be due to the low hydroxyproline content in sea cucumber collagen which has a direct influence on creating the three-dimensional branched network during gel-formation [120,206].

In addition, hydrolysis may occur in some amide bonds in the primary chain of collagen molecules during the gelation process [8]. The gelation process of collagen, as well as gelatin, are referred as thermo reversible processes [9]. Gel strength and gel melting point are significant physical properties of gelatin gels [8]. The melt-in-the-mouth property of gelatin is considered as one of the significant characteristics of gelatin, which is extensively utilized by both food and pharmaceutical industries [8].

Hydrophilic nature and swelling ability of solubilized collagen are used to minimize the dripping loss of frozen fish and meat products [207]. Moreover, for enhancing the sensory characteristics, collagenous materials are used widely in the food industry due to their gelling properties [208]. Apart from that, collagen and gelatin are utilized as wetting agents in food, pharmaceutical, and medical applications [8,9].

Dong et al. [115] studied the changes of collagen in sea cucumber *Stichopus japonicas* during cooking and reported that thermal treatments on the sea cucumber affect the appearance and the sensory properties of the final product. This is due to the alteration of water absorption ability of collagen. Zhu et al. [95] investigated the moisture absorption and retention capacities of PSC from sea cucumber and suggested that PSC might be an excellent functional ingredient for cosmetics as they exhibited a behavior comparable to that of glycerol. Li et al. [83] investigated the collagen from sea cucumber *Holothuria cinerascens* and evaluated its potential application in moisturizing cosmetic products. They reported that the polar groups, including carboxyl (-COOH) and hydroxyl (-OH) groups on the surface of the collagen molecule, promote the moisture retention of products.

### 7.2. Emulsifying Properties

Charged groups of collagens contain hydrophilic or hydrophobic amino acids that are responsible for its surface properties. In an aqueous system, hydrophobic and hydrophilic groups are involved in reducing surface tension by moving to the surface area of the emulsion [209,210]. Hydrophobic areas on the peptide chain have a major impact on the emulsifying and foaming properties of gelatin [209].

In addition, surface-active property and gel firmness are other crucial factors affecting emulsion properties. The emulsion capacity is increased with protein concentration [9]. In addition, molecular weight also influences the stabilization of the emulsion, as high-molecular-weight gelatin forms a more stable emulsion compared to low-molecular-weight one [9,211]. Moreover, factors like temperature, pH, concentration, and homogenization of the collagen may also affect the emulsifying and foaming properties of collagen [212]. Higher content of hydrophobic amino acid favors increased foam capacity of gelatin [211,212].

Furthermore, the stability of foams depends on various parameters including the rate of attaining equilibrium surface tension, bulk and surface viscosities, steric stabilization, and electrical repulsion between the two sides of the foam lamella [211].

### 7.3. Film Forming Properties

Biodegradable films made from edible protein-based biopolymers are gaining popularity in the food industry due to consumers’ awareness and their low impact on the environment [213,214]. However, the hygroscopic nature of gelatin limits its use as a protective barrier [9] and usually following the extraction process, collagen molecules tend to lose their mechanical properties compared to the native form [214]. Several investigations have been carried out to improve the mechanical and water resistance properties of these films with the addition of other biopolymers such as chitosan, hydrophobic and hydrophilic plasticizers, lipids, and protein isolates, among others [9,214].

Avena–Bustillos et al. [215] studied the water vapor permeability of mammalian and fish gelatin films and found lower water permeability in fish gelatin films compared to those from mammalian sources. Moreover, water vapor permeability of cold-water and warm-water fish gelatin are also different as warm-water fish always exhibits a higher water permeability compared to that of cold-water fish gelatin. However, excellent film-forming property of fish gelatin expands its usage in encapsulated drugs and frozen foods. The hydrophobicity of the protein is also an essential factor for its film formation. Notably, low hydrophobicity of marine collagen may be due to a lesser availability of proline and hydroxyproline for hydrogen bonding with water [9].

Furthermore, the film-forming ability of collagen and collagen-based derivatives like gelatin depends on their molecular weight distribution and amino acid composition that can directly affect the mechanical and barrier properties of films [216]. Recent research has been focusing on enriching these films with the addition of antioxidants and antimicrobial substances to enhance their application as a renewable biomaterial [217].

## 8. Challenges and Future Perspectives of Sea Cucumber Collagen

Collagen as a biomaterial is now moving towards addressing certain limitations related to its inconsistent production to meet the industrial requirement. Due to their unique characteristics, including biocompatibility and other physicochemical properties, collagens are not easily substituted by other molecules and finding alternatives might be a difficult task. Identifying new natural sources of collagen and upgrading the existing methodologies for extraction, isolation, and purification can be effective alternative solutions to overcome the existing challenges.

In this scenario, marine collagen emerges as a potential alternative source to fulfill the increasing demand of natural collagen from other sources. Owing to its excellent biocompatibility, low risk of transmissible diseases, no or low ethical and religious constraints, marine derived collagen has been recognized as a promising source of pharmaceutical and food grade commodity. Among the various sources of marine organisms, sea cucumber is identified as a potent, yet underexploited, source of collagen. However, collagen from marine sources contributes less than 1.5% to the total collagen production [9,10]. Marine animal collagens are considered as being relatively low-quality due to their poor rheological properties and thermal stability, mainly dictated by their amino acid compositions which depend on the environmental and body temperature of aquatic animals. Hence, the techno-functional feasibility of commercialization of collagen and collagen peptides derived from sea cucumber may face many challenges. Existing clinical trials on the bio-efficacy of sea cucumber derived collagen and its derivatives are inadequate. Therefore, further exploration of functional activities of sea cucumber derived collagen and hydrolysates thereof is urgently needed to overcome these hurdles. Moreover, it is always crucial to consider consumer acceptance, especially when incorporating collagen and its derivatives into functional food products. Usually, low-molecular-weight peptides (contain amino acid residues) may impart a bitter taste to products, hence may adversely affect their sensory attributes. Besides, the cost associated with the product is also a significant factor in gaining consumer acceptance for innovative products. Therefore, it is vital to consider consumer’s perspectives before launching a new product.

In addition, issues in the commercialization process of marine-derived collagen can be resolved by developing strategies to full utilization of the marine resources. Use of marine by-products (discards) for the extraction of value-added products like collagen would be an ideal approach to maximize the sustainability and economic viability of the industry. It is also essential to consider the reproducibility of collagen extraction along with economic viability in an industrial scale. Furthermore, before commercializing marine-based collagen and its derivatives, it is mandatory to consider the market potential, competition, overall production costs, and business environment. Therefore, efforts should be directed towards exploring sea cucumber, one of the underutilized marine resources, as a potential source of high-value collagen peptides. Further research is needed to focus on the implementation of novel technologies for extraction, isolation, purification and characterization of sea cucumber derived collagen and their derivatives for maximizing the yield, recovery, and purity of collagen with less impact on the environment.

## Figures and Tables

**Table 1:** Common types of collagen.

Collagen Type	Chains	Sub Family	Distribution
**I**	α1(I) α2(I)	Fibrillar collagen	Skin, tendon, bone, dermis, intestine, uterus
**II**	α1(II)	Fibrillar collagen	Hyaline cartilage, vitreous, nucleus pulposus
**III**	α1(III)	Fibrillar collagen	Dermis, intestine, large vessels, heart valve
**IV**	α1(IV) α2(IV) α3(IV) α4(IV) α5(IV) α6(IV)	Basement membrane and associated collagen	Basement membranes
**V**	α1(V) α2(V) α3(V)	Fibrillar collagen	Cornea, placental membranes, bone, large vessels
**VI**	α1(VI) α2(VI) α3(VI)	Beaded filament forming collagen	Descement’s membrane, skin, heart muscles
**VII**	α1(VII)	Basement membrane and associated collagen	Skin, placenta, lung, cartilage, cornea
**VIII**	α1(VIII) α2(VIII)	Short chain collagen	Produced by endothelial cells, descemet’s membrane
**IX**	α1(IX) α2(IX) α3(IX)	Fibril associated and related collagen	Cartilage
**X**	α1(X)	Short chain collagen	Hypertrophic and mineralizing cartilage
**XI**	α1(XI) α2(XI) α3(XI)	Fibrillar collagen	Cartilage, intervertebral disc, vitreous humor
**XII**	α1(XII)	Fibril associated and related collagen	Chicken embryo tendon, bovine periodontal ligament
**XIII**	α1(XIII)	Trans membrane collagens and collagen like proteins	Cetal skin, bone, intestinal mucosa

Source: Adapted from [40,41].

**Table 2:** Alternative land animal sources for bovine and porcine collagen.

Source	Extraction Method	Purpose of Extraction	Reference
Chicken feet	Acid extraction	Optimization of extraction condition	[43]
	Enzyme extraction	Determination of pepsin digestion effect on the properties of extracted collagen	[48]
	Acid extraction	Preparation of edible films	[57]
	Enzyme extraction (using papain and pepsin)	Isolation and characterization of chicken feet originated collagen	[58]
	Acid extraction	Use of chicken feet for protein films	[59]
	Alkali, acid, and enzyme extraction	Identification of best method of collagen extraction method and characterization of chicken feet collagen	[50]
	Enzyme extraction	Optimization of extraction process and synthesis of chicken feet collagen based biopolymeric fibers	[60]
Rat tail tendon	Acid extraction	Preparation of type I collagen for tissue engineering applications	[45]
Alligator bone	Acid and enzyme assisted extraction	Determination of biochemical properties of alligator bone collagen	[47]
Silky fowl feet	Combination of acid and enzyme extraction	Identification of best combination for high quality collagen extraction method	[49]
Ovine tendon	Acid extraction	Determination of the biocompatibility of ovine tendon originated collagen with human dermal fibroblast	[51]
	Acid extraction	Determination of the biocompatibility of ovine tendon originated collagen with human dermal fibroblast Improve the mechanically strong ovine tendon originated collagen for tissue engineering purposes	[52]
	Acid extraction	Characterization and fabrication of thin films from ovine tendon collagen for tissue engineering applications	[53]
	Acid extraction	Investigation of attachment, proliferation, and morphological properties of human dermal fibroblasts on ovine tendon collagen	[54]
Duck feet	Acid extraction	Investigation of physicochemical properties of collagen derived from duck feet	[46]
	Acid extraction	Determination of feasibility of using duck feet collagen in improving physicochemical properties of surimi	[61]
Kangaroo tail	Acid extraction	Identification of alternative collagen sources for pre-clinical models for cell biology	[44]
Sheep bone	Acid extraction	Determination of effect of different collagen extraction protocols	[55]
Equine tendon	Acid extraction	Evaluation of the effects of different extraction methods on the collagen structure of equine tendons	[33]

**Table 3:** Recent studies on sea cucumber collagen.

Sea Cucumber Species	Focus of Study	Major Findings	Reference
*Stichopus japonicus*	Chemical composition and subunit structure of collagen	Collagen was comprised of 2 distinct subunits (α1 and α2 and rich in glutamic acid compared to other fibrillar collagen	[14]
	Characterization and subunit composition of collagen	Pepsin solubilized collagen resembled type I collagen and its amino acid composition was different from vertebrate collagen.	[16]
	Changes of collagen during cooking	Crude collagen fibers were more susceptible to heat treatment compared to pepsin-solubilized collagen	[115]
	Identification of physicochemical properties and radical scavenging capacities of pepsin-solubilized collagen	Extracted collagen maintained intact triple-stranded helices and high moisture retention and absorption capacities as well as exhibiting better radical scavenging ability compared to vitamins C and E.	[95]
	Wound-healing effects on human keratinocyte (HaCaT) cell line of pepsin-solubilized collagen	Pepsin-solubilized collagen has the potential to use as an alternative mammalian collagen in the nutraceutical and pharmaceutical industries	[96]
	Effect of autolysis of intact collagen fibers related to the distributions of cathepsin L	Lysosomal cathepsin L degrades the collagen fibers and speed and degree of autolysis is negatively correlated with the density of collagen.	[116]
	Structural characteristics of sea cucumber collagen fibers in the presence of endogenous cysteine proteinases	Collagen fibrils disaggregated into collagen fibrils by cysteine proteinases and the structural disorder of the native collagen fibers increased due to cysteine protease.	[102]
	Structural and biochemical changes of collagen related to autolysis	Collagen fibers and microfibrils gradually degraded with the autolysis and structural damage was less in monomeric collagen compared to other structural elements	[103]
	Structural and thermal properties of sea cucumber collagen	Distance between adjacent molecular chains in collagen molecules was decreased and CO_2_, NH_3_, H_2_O, CH_4_, NO_2_ and HCN gases released during the heat treatment	[117]
	Enzymatic hydrolysis of collagen to determine the structural changes of collagen fibrils	Collagen fibers were partially degraded into collagen fibrils by enzymatic (trypsin) treatments	[118]
	Investigate the effect of collagenase type I on the structural features of collagen fibers	Collagenase was responsible for partial depolymerization of collagen fibers into fibrils, uncoiled the fibrils, degrade monomeric collagen	[119]
*Parastichopus californicus*	Purification and characterization of pepsin-solubilized collagen from skin and connective tissue	Collagen extracted from skin and connective tissue contains type I collagen with three α1 chain. Amino acid composition is different from the mammalian type I collagen	[120]
*Bohadschia* spp.	Analysis of isolated pepsin-solubilized collagen	Type I collagen was identified with three α1 chain	[121]
*Stichopus vastus*	Isolation and characterization of pepsin-solubilized collagen	Purified collagen belongs to type I collagen contains three α1 chain with triple helical structure	[99]
	Molecular mass distribution, amino acid composition and radical-scavenging activity of collagen hydrolysates prepared from isolated collagen	β and α1 chains of the collagen were hydrolyzed by trypsin and molecular mass distribution ranged from 5 to 25 kDa. Hydrolysates contains high glycine, alanine, glutamate, proline and hydroxyproline residues and showed significant radical scavenging activity	[122]
	Physicochemical and biochemical properties of pepsin solubilized collagen	Glycine was the predominant amino acid present in purified collagen that possessed high moisture absorption and retention capacity	[122]
	Identification of Angiotensin I converting enzyme (ACE) inhibitory and radical scavenging activities from collagen hydrolysates	Novel bioactive peptides generated by optimized trypsin hydrolysis have the potential to use as ACE inhibitors and radical scavenging agents.	[100]
*Holothuria parva*	Purification and characterization of pepsin-solubilized collagen	Isolated collagen constituted three α1 chain and was rich in glycine, proline, alanine and hydroxyproline	[98]
*Stichopus monotuberculatus*	Isolation and characterization of pepsin-solubilized collagen	Isolated collagen was classified as type I collagen consisted of three α1 chain	[101]
*Holothuria scabra*	Determination of nano-collagen quality and extraction of acid solubilized collagen	Extracted acid solubilized collagen had significant effect on physicochemical characteristics of nano-collagen particles	[123]
*Australostichopus mollis*	Biochemical composition of isolated collagen	Type I collagen was present with α1 and α2 chains, α chain dimers, β chains, and γ components. Most abundant amino acids were glycine, alanine, threonine, serine, and proline.	[124]
*Holothuria leucospilota*	In vitro activity of anti-tyrosinase and anti-elastase activity of isolated collagen	Isolated collagen exhibited weak anti-tyrosine activity and moderate anti-elastase activity	[125]
*Acaudina leucoprocta*	Extraction methods to remove heavy metals from the isolated collagen	Pepsi- solubilized collagen showed two isoforms and amount of heavy metals present in the collagen were below the contaminant limit	[126]
*Acaudina molpadioides*	Preparation and characterization of antioxidative peptides from collagen hydrolysates	Collagen peptides which showed highest antioxidant activity were rich in hydrophobic acid residues.	[127]
*Stichopus vastus and Holothuria atra*	Comparison of partial characteristics of two different sea cucumbers	No significant difference in amino acid composition, yield, or whiteness	[128]
*Apostichopus japonicus*	Type of constituent collagen using proteomics and bioinformatic strategies	Heterogenicity of the sea cucumber collagen fibrils was revealed for the first time that provides novel insight into the composition of sea cucumber collagen	[104]
	Analysis of the effect of epigallocatechin gallate (EGCG) on preserving molecular structure of collagen fibers during heating	EGCG protects the structure of crude collagen fibers in a dosage dependent manner and effects hydrogen bonds on the collagen which promotes protein aggregation	[105]
*Holothuria cinerascens*	Potential application of collagen in moisturizing cosmetics	Collagen showed better moisture retention and moisture absorption capacity. Abundant hydrophilic groups in collagen increases their ability for cosmetic formulations	[83]

**Table 4:** Distinct characteristics of sea cucumber collagen compared to mammalian collagen.

Characteristics	Sea Cucumber Derived Collagen	Mammalian Collagen	Reference
Abundant type	Type I collagen	Type I collagen	[14,18,113]
Differences in amino acid composition	Low hydroxyproline content, high glutamic and aspartic acid residues	High hydroxyproline content, low glutamic acid and aspartic acid residues	[14,16,99,101,120]
Covalent cross links	Internally present and provide stabilization to the molecule	Internally present and provide stabilization to the molecule	[109,110,111]
Thermal stability	Low thermal stability with low denaturation temperature compared to mammalian collagen	High thermal stability compared with high denaturation temperatures	[98,101,120,122]
Resistance to protease digestion	Relatively low	Relatively high	[99]
Gel forming ability	Comparatively low	Comparatively high	[99]
Moisture absorption ability	Relatively high	Relatively high	[8,95]

**Table 5:** Pre-treatment procedures and methods used for sea cucumber collagen identification.

Sea Cucumber Species	Body Parts	Pre-Treatment	Methods Used for Characterization of Collagen	Reference
*Cucumaria frondosa*	Inner dermis	Incubation with deionized water	Amino acid analysis SDS-PAGE Salt solubility determination	[109]
*Stichopus japonicus*	Body wall	Disaggregation with β-mercaptoethanol and 0.1 M NaOH treatment	Amino acid analysis SDS-PAGE DSC	[14]
	Body wall	Incubation with water	Ultraviolet-visible (UV-vis) spectra SDS-PAGE Peptide mapping Amino acid composition DSC Gel filtration chromatography	[16]
*Stichopus vastus*	Integument	Incubated with water	UV-vis spectra SDS-PAGE peptide mapping FTIR Gel forming capacity	[99]
*Bohadshia* spp.	Body wall	Washed in distilled water	SDS-PAGE	[165]
*Holothuria parva*	Skin	Washed in distilled water	SDS-PAGE DSC Gel-forming capacity UV-vis spectra Amino acid composition Scanning electron microscopy	[98]
*Stichopus monotuberculatus*	Body wall	Homogenization with water	UV-vis spectra SDS-PAGEA mino acid analysis FTIR Enzyme-digested peptide mapping DSC Solubility level	[142]
*Parastichopus californicus*	Skin and connective tissue	Washed in distilled water	DSC SDS-PAGE Enzyme-digested peptide mapping Gel-forming capability Amino acid composition	[120]
*Australostichopus mollis*	Body wall	Washed in distilled water	Scanning electron microscopy Electrophoretic analysis Peptide mapping UV-vis spectra DSC FTIR Amino acid analysis	[124]
*Acaudina molpadioides*	Body wall	Soaked in 0.2 M EDTA for 48 h	Gel-filtration chromatography Amino acid analysis RP-HPLC and identification of peptide sequence	[127]

**Table 6:** Sodium dodecyl sulphate polyacrylamide gel electrophoresis (SDS-PAGE) analysis methods conducted on different sea cucumber species.

Sea Cucumber Type	SDS Gel Composition	Collagen Type and Subunit Composition	Findings	Reference
*Cucumaria frondosa*	Linear polyacrylamide gradients of 4–20%, and 100 mM Tris, 3.3% SDS, 20% glycerol	Type I collagen (α1)_3_	Covalent composition of collagen is α1 trimer and amino acid composition is similar to human collagen type I	[109]
*Stichopus japonicus*	Consisted with 9% polyacrylamide gels	Type I collagen, consisting of 1 α trimer (approximately 135 kDa)	Subunit structure of isolated collagen is similar to (α1)_3_ pattern that exists in the invertebrate collagen	[16]
*Parastichopus californicus*	Discontinuous Tris-HCl/glycine buffer system with 7.5% resolving gel and 4% stacking gel	Type I collagens, consisting of three α1 chains of approximately 138 kDa	Isolated collagen constituents were α1 and β dimers and similar to that reported for collagens from other sea cucumber species	[120]
*Stichopus japonicus*	Discontinuous Tris-HCl/glycine buffer system with 10% separating gel and a 5% stacking gel	Type I collagens, consisting of 1 α trimer	Electrophoresis pattern demonstrated a major single band on SDS-PAGE	[105]
*Stichopus vastus*	Discontinuous Tris-HCl-glycine buffer system with 75 g L^−1^ resolving gel and 40 g L^−1^ stacking gel	Type I collagen, consisting of three α1 chains of approximately 122 kDa each	Isolated collagen was consisted with major component (α1) of approximately 122 kDa and a small amount of β dimers (about 267 kDa each) similar to that reported for collagen from other sea cucumber species	[99]
*Bohadschia* spp.	Discontinuous Tris-HCl-glycine buffer system with 7.5% resolving gel and 4% stacking gel	Type I collagen with three α1 chains with approximately 138 kDa each	Collagen was formed with major component of α1 and smaller amount of β dimer	[165]
*Stichopus monotuberculatus*	Discontinuous tris-glycine buffer system electrophoresis with 7.5% precast gel	Type I collagen consists of three α1 with molecular weight of 137 kD	Collagen consisted of 3 homologous α1 chains as (α1)_3_. The molecular weight of isolated collagen was similar to the reported values of collagens from other species	[101]
*Australostichopus mollis*	Not included in detail	Type I collagens consist of α1 and α2 chains (approximately 116 kDa)	Collagen formed α1 and α2 chains with α chains dimer, β chains (around 212 kDa) and small amounts of γ components and electrophoresis pattern was similar to those of calf skin collagen	[124]
*Holothuria cinerascens*	10% SDS separating gel and 5% stacking gel	Type I collagen with identical α1 chains (α1, α2 and α3)	Molecular weight of isolated α chains extracted was about 80–90 kDa, and the molecular weight of the β-chain was about 150–160 kDa. The reported molecular weights were significantly lower than those of tilapia and porcine skin collagen	[83]
